# Arabidopsis Fructokinases Are Important for Seed Oil Accumulation and Vascular Development

**DOI:** 10.3389/fpls.2016.02047

**Published:** 2017-01-10

**Authors:** Ofer Stein, Tamar Avin-Wittenberg, Ina Krahnert, Hanita Zemach, Vlada Bogol, Oksana Daron, Roni Aloni, Alisdair R. Fernie, David Granot

**Affiliations:** ^1^Volcani Center, Institute of Plant Sciences, Agricultural Research OrganizationBet Dagan, Israel; ^2^Robert H. Smith Faculty of Agriculture, Institute of Plant Sciences and Genetics in Agriculture, Food and Environment, Hebrew University of JerusalemRehovot, Israel; ^3^Max-Planck-Institut für Molekulare PflanzenphysiologiePotsdam-Golm, Germany; ^4^Department of Plant and Environmental Sciences, Hebrew University of JerusalemGivat Ram, Jerusalem, Israel; ^5^Department of Life Sciences, Ben-Gurion UniversityBeer-Sheva, Israel; ^6^Department of Plant Sciences, Tel Aviv UniversityTel Aviv, Israel

**Keywords:** fructokinase, seed oil, xylem, fatty acid, carbon metabolism

## Abstract

Sucrose (a disaccharide made of glucose and fructose) is the primary carbon source transported to sink organs in many plants. Since fructose accounts for half of the hexoses used for metabolism in sink tissues, plant fructokinases (FRKs), the main fructose-phosphorylating enzymes, are likely to play a central role in plant development. However, to date, their specific functions have been the subject of only limited study. The Arabidopsis genome contains seven genes encoding six cytosolic FRKs and a single plastidic FRK. T-DNA knockout mutants for five of the seven FRKs were identified and used in this study. Single knockouts of the FRK mutants did not exhibit any unusual phenotype. Double-mutants of *AtFRK6* (plastidic) and *AtFRK7* showed normal growth in soil, but yielded dark, distorted seeds. The seed distortion could be complemented by expression of the well-characterized tomato *SlFRK1*, confirming that a lack of FRK activity was the primary cause of the seed phenotype. Seeds of the double-mutant germinated, but failed to establish on 1/2 MS plates. Seed establishment was made possible by the addition of glucose or sucrose, indicating reduced seed storage reserves. Metabolic profiling of the double-mutant seeds revealed decreased TCA cycle metabolites and reduced fatty acid metabolism. Examination of the mutant embryo cells revealed smaller oil bodies, the primary storage reserve in Arabidopsis seeds. Quadruple and penta FRK mutants showed growth inhibition and leaf wilting. Anatomical analysis revealed smaller trachea elements and smaller xylem area, accompanied by necrosis around the cambium and the phloem. These results demonstrate overlapping and complementary roles of the plastidic AtFRK6 and the cytosolic AtFRK7 in seed storage accumulation, and the importance of AtFRKs for vascular development.

## Introduction

Sucrose, a disaccharide, is an important end product of photosynthesis and the primary carbon source for metabolism in sink tissues of many plants, including Arabidopsis. Sucrose must be cleaved by either sucrose synthase (SUS) into UDP-glucose and fructose, or by invertase into glucose and fructose, before it can be further metabolized (Dennis and Blakeley, 2000). The free hexoses, fructose and glucose, must then be phosphorylated by fructokinase (FRK) or hexokinase (HXK) before they can enter metabolic pathways. FRK and HXK are distinguished by their substrate specificities and affinities (Renz and Stitt, 1993; Dai et al., 2002; Granot, 2007). FRK phosphorylates only fructose; whereas HXK phosphorylates both glucose and fructose. However, the affinity of FRK for fructose is two orders of magnitude higher than that of HXK for fructose (Granot, 2007). It is, therefore, likely that fructose is phosphorylated primarily by FRKs, making those enzymes key players in the primary metabolism and development of plants.

Several FRKs have been identified in a number of plant species including tomato (*Solanum lycopersicum*), potato (*Solanum tuberosum*), maize (*Zea mays*), soybean (*Glycine max*), barley (*Hordeum vulgare*), spinach (*Spinacia oleracea*) and pea (*Pisum sativum*) (Pego and Smeekens, 2000). In tomato, for example, currently the best characterized plant species with regard to FRK, four FRK genes have been identified (*SlFRK1-4*) encoding enzymes with different intracellular localization and biochemical characteristics. Three of the tomato FRK enzymes are located in the cytosol and a single tomato FRK (SlFRK3) is found in plastids (Kanayama et al., 1997, 1998; German et al., 2002, 2004; Damari-Weissler et al., 2006; Granot, 2007). FRKs are expressed at different levels in almost all plant tissues, yet, in tomato, *SlFRK4* is expressed specifically in stamens and pollen (German et al., 2002, 2003, 2004; David-Schwartz et al., 2013; Granot et al., 2013).

Increasing evidence suggest that FRKs are important for vascular development. The tomato *SlFRK2* is essential for proper xylem development, and the xylem vessels in stems of *SlFRK2*-antisense plants have thinner xylem secondary cell walls and those cells are narrower and deformed (Damari-Weissler et al., 2009). As a result, water conductance is reduced, causing severe growth inhibition and the wilting of young leaves (Damari-Weissler et al., 2009). The tomato plastidic FRK is also important for xylem development, as indicated by the fact that RNAi suppression of *SlFRK3* decreases plant hydraulic conductivity and transpiration. Suppression of both the cytosolic *SlFRK2* and the plastidic *SlFRK3* yielded deformed xylem vessels and fibers with thin cell walls, implying that both genes play a role in xylem fiber development (Stein et al., 2016). FRK is also important for xylem fiber development in aspen wood (*Populus tremula x tremuloides*), in which the suppression of the cytosolic *FRK2* yielded narrower xylem fibers perhaps due to a decrease in cellulose content (Roach et al., 2012).

To date, only two FRK proteins have been identified in Arabidopsis by native polyacrylamide gel electrophoresis followed by activity staining (Gonzali et al., 2001). However, the genes that code for these two proteins have not yet been identified. The Arabidopsis genome contains seven genes of the pfkb-family protein, which are predicted to be FRKs. The aim of this work was to identify and characterize T-DNA mutants for the genes encoding Arabidopsis FRKs and to investigate their importance for plant development.

## Materials and methods

### Plant material, growth conditions, and sugar treatments

Arabidopsis (*Arabidopsis thaliana*) wild-type plants, T-DNA-tagged mutant plants and transgenic plants used in this work were of the Col-0 ecotype. Mutant seeds with a T-DNA insertion in their *AtFRK* genes were obtained from the Arabidopsis Biological Resource Center and are listed in Table S1. Seeds were sown in soil or sterilized and sown on half-strength Murashige and Skoog (MS) medium (Murashige and Skoog, 1962) with or without 1% sucrose, glucose, fructose or mannitol. Seeds were kept at 4°C for 3 d in the dark for stratification and then transferred to normal growth conditions. Plants were grown in a walk-in growth chamber kept at 22°C with a light intensity of 80 μmol m^−2^ s^−1^ and a 16-h light/8-h dark photoperiod unless stated otherwise.

### Vector construction and plant transformation

The FRK1 cDNA (SlFRK1) from tomato (*Solanum lycopersicum* L.; GenBank accession number U64817) was inserted in the sense orientation between the cauliflower mosaic virus 35S promoter and the nopaline-synthase termination site in the binary vector pBI121 (Odanaka et al., 2002). The beta-glucuronidase gene in pBI121 was removed by digestion with BamHI and SacI, and was replaced with FRK1 cDNA including ~270 bp of the 5′ untranslated region and ~50 bp of the 3′ untranslated region. This FRK1 vector was introduced into *Agrobacterium tumefaciens* for the transformation. Agrobacterium-mediated transformation of *Arabidopsis thaliana* was performed using the floral-dip method as described previously (Clough and Bent, 1998).

### Seed weight

Seeds of the WT, *frk6, frk7*, and the double-mutant were harvested from plants grown under even-day conditions (12-h photoperiod) at the same time. One thousand two hundred to one thousand eight hundred seeds from three individual plants per line were photographed on a white paper and counted manually using ImageJ software (http://rsb.info.nih.gov/ij/). The seeds were then weighed using an analytical scale and that figure was divided by the number of seeds per plant to determine the average seed weight per plant.

### DNA extraction, RNA extraction, cDNA preparation, and PCR analysis

Genomic DNA was extracted as described previously (Edwards et al., 1991). Genotyping was done with right plus left primers flanking the T-DNA of each of the SALK lines and with right primer plus the LBb1 primer for confirming existence of the T-DNA insertion. Primers used for PCR amplification are listed in Table S2.

Total RNA was extracted using the LogSpin method (Yaffe et al., 2012). Samples were ground using a Geno/grinder (SPEX SamplePrep, Metuchen, NJ, USA) and RNA was extracted in 8 M guanidine hydrochloride buffer (Duchefa Biochemie, Haarlem, The Netherlands) and transferred to tubes containing 96% EtOH (Bio Lab, Jerusalem, Israel). Then, samples were transferred through a plasmid DNA extraction column (RBC Bioscience, New Taipei City, Taiwan), followed by two washes in 3 M Na-acetate (BDH Chemicals, Mumbai, India) and two washes in 75% EtOH and eluted with DEPC (diethylpyrocarbonate) water (Biological Industries Co., Beit Haemek, Israel) that had been preheated to 65°C. The RNA was treated with RQ1-DNase (ProMega, Madison, WI, USA) according to the manufacturer's instructions, to degrade any residual DNA. For the preparation of cDNA, total RNA (1 μg) was taken for reverse transcription-PCR using MMLV RT (ProMega) in a 25-μl reaction, with 2 μl of random primers (ProMega) and 1 μl of oligo-dT primers (ProMega). cDNA samples were diluted 1:4 in water. Amplification of the AtFRKs was done by PCR. Following an initial preheating step at 94°C for 2 min, there were 37 cycles of amplification consisting of 20 s at 94°C, 20 s at 60°C, and 60 s at 72°C. The Arabidopsis *AtHXK1* (accession no. At4g29130) was used as a reference gene. Primers used for PCR amplification are listed in Table S2.

### Scanning electron microscopy (SEM)

Dry seeds were attached to a metal stub with double-sided carbon tape and coated with gold palladium (Quorum SC7620 mini sputter coater). Images were taken with a JEOL JCM-6000 benchtop SEM. Analysis was performed using SEM software.

### Extraction, derivatization, and analysis of arabidopsis seeds primary metabolites using GC-MS

For each line, 40 mg of dry Arabidopsis seeds from six individual plants were carefully cleaned of debris and collected in 2-ml Eppendorf tubes. The samples were frozen in liquid nitrogen and ground using a Geno/grinder (SPEX SamplePrep, Metuchen, NJ, USA). The samples were extracted in 1 mL of methanol/chloroform/DDW solution (2.5/1/1) and 15 μl internal standard was added (0.2 mg ml^−1^ ribitol in water). Following 1 h of shaking at 4°C, the samples were centrifuged for 10 min at 20,800 *g* and 900 μl of the supernatant were transferred to a new 1.5-ml tube. Five hundred microliter DDW were added for phase separation and the upper polar phase was transferred to a new 1.5-ml tube and dried using a speed-vac before storage. Derivatization, standard addition and sample injection were exactly as described previously (Lisec et al., 2006).

The GC-MS system was comprised of a CTC CombiPAL autosampler, an Agilent 6890N gas chromatograph and a LECO Pegasus III TOF-MS running in EI+ mode. Metabolites were identified in comparison to database entries of authentic standards (Kopka et al., 2005). Chromatograms and mass spectra were evaluated using Chroma TOF 1.0 (LECO) and TagFinder 4.0 software (Luedemann et al., 2008). Data presentation and experimental details are provided as supplemental data in a manner consistent with recent metabolite reporting recommendations (Araújo et al., 2011) (Supplemental Data file 1).

### Fatty acid (fames) extraction and measurement using gas chromatography coupled to a flame ionization detector (GC-FID)

For each line, 20 dry Arabidopsis seeds from six individual plants were counted and carefully weighted using analytical scales. Seed fatty acids were extracted exactly as described previously (Focks and Benning, 1998). Briefly, seeds were homogenized and incubated with 1 ml 1 N HCl in methanol. To each sample, 100 μl of internal standard (FA15:0, pentadecanoic acid) were added. Samples were then incubated at 80°C in a water bath for 30 min. After the vials had cooled down to the room temperature, 1 ml of 0.9% NaCl and 1 ml 100% hexane were added to each vial. Vials were shaken for 5 s and centrifuged for 4 min at 1000 rpm. The upper FAMEs-containing hexane phase was transferred to a new glass vial, where it was concentrated in an N_2_ stream. Finally, FAMEs were dissolved in hexane and poured into GC glass vials. The GC-FID method data: injector temperature: 250°C; carrier gas: helium; head pressure: 25 cm s^−1^, 11.8 psi; GC-column J&W DB23 (Agilent), 30 m × 0.25 mm × 0.25 μm; detector: 250°C; detector gas: H_2_ 40 ml min^−1^, air 450 ml min^−1^, He make-up gas 30 ml min^−1^.

### Statistical analysis

Relative metabolite and FA levels were obtained as ratios between the lines and the mean value of the respective wild type. Statistical differences between groups were analyzed using Student's *t*-tests. Results were determined to be statistically different at a probability level of *P* < 0.05. The calculated ratios were subjected to a principal component analysis (PCA) performed using the Multibase Excel add-in (Numerical Dynamics).

### Transmission electron microscopy (TEM)

Dry Arabidopsis Col-0 and *frk6 frk7* seeds were placed on adhesive tape and a small portion of the seed was cut out with a sharp knife, in order to allow processing material easy access to the embryo. The seeds were fixed in 5% glutaraldehyde in 0.1 M cacodylate buffer (pH 7.4) overnight at room temperature. The tissues were then rinsed four times, 10 min each rinse, in cacodylate buffer and post-fixed and stained with 2% osmium tetroxide, 1.5% potassium ferricyanide in 0.1 M cacodylate buffer for 1 h. Tissues were then washed four times in cacodylate buffer and dehydrated in increasing concentrations of ethanol (i.e., 30, 50, 70, 80, 90, and 95%),10 min each step, followed by three 20-min dehydrations in 100% anhydrous ethanol and two 10-min dehydrations in propylene oxide. Following dehydration, the tissues were infiltrated with increasing concentrations of Agar 100 resin (i.e., 25, 50, 75, and 100%) in propylene oxide, with each step lasting 16 h. The tissues were then embedded in fresh resin and left to polymerize in an oven at 60°C for 48 h.

Embedded tissues in blocks were sectioned first with a glass knife (2 μm) and stained with methylene blue, and then with a diamond knife on an LKB 3 microtome and ultrathin sections (80 nm) were collected onto 200-mesh, carbon/formvar-coated copper grids. The sections on grids were sequentially stained with uranyl acetate and lead citrate for 10 min each and viewed with Tecnai 12 TEM 100 kV (Phillips, Eindhoven, the Netherlands) equipped with MegaView II CCD camera and Analysis software, version 3.0 (SoftImaging System GmbH, Münstar, Germany).

### Anatomical techniques

Free-hand cross-sections were taken from 8-week-old WT, quadruple-mutant and penta-mutant plants as shown in **Figure 8**. The cross-sections were stained for a few seconds in 2% lacmoid in 96% ethanol and then rinsed in tap water for a few minutes, mounted in 50% sodium lactate (Aloni, 1980) and observed under transmitted white light.

## Results

### Identification of arabidopsis FRK genes

**Figure 1:**
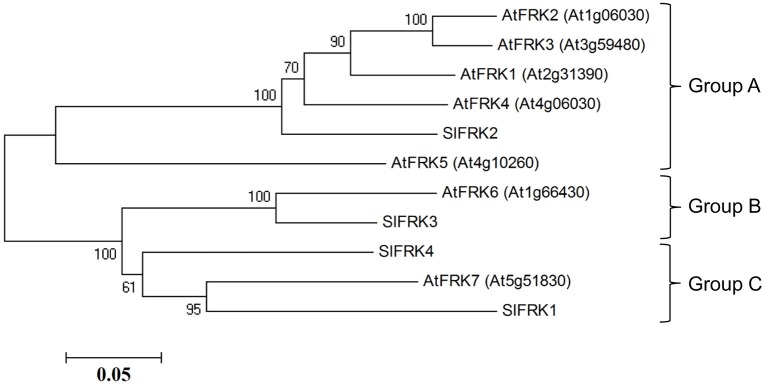
**Phylogenetic relationships between tomato and Arabidopsis fructokinases**. Phylogenetic evolutionary analysis was conducted using *MEGA* version 4 (Tamura et al., 2007). This phylogenetic tree is based on the following protein sequences: SlFRK1 (NP_001233893), SlFRK2 (NP_001233888), SlFRK3 (NP_001234396), SlFRK4 (NP_001234206), At5g51830 (NP_199996), At1g66430 (NP_564875), At4g10260 (NP_192764), At3g59480 (NP_191507), At1g06030 (NP_172093), At1g06020 (NP_172092), and At2g31390 (NP_180697).

Protein blast analysis (http://blast.ncbi.nlm.nih.gov/Blast.cgi) with the four characterized tomato FRK proteins against Arabidopsis proteins identified seven genes from the pfkb protein family denoted as probable FRK1-7 by the uniprotKB/swissprot plant proteome annotation program (Schneider et al., 2009). A phylogenetic tree created from Arabidopsis and tomato FRKs proteins (Figure 1) showed that one protein (At1g66430, assigned FRK6) is homolog of the plastidic *SlFRK3* and that another, Arabidopsis FRK (At5g51830, assigned FRK7), is closely related to *SlFRK1*, while five other proteins (At2g31390, At1g06030, At1g06020, At3g59480, At4g10260 assigned FRK1-5, respectively) are closely related to *SlFRK2*, the main cytosolic FRK in tomato. Although a comparison of their respective sequences does not suggest any relation, it appears that *AtFRK5* (At4g10260) might be functionally related to *SlFRK4* as both are expressed exclusively in pollen (David-Schwartz et al., 2013). Prediction of the subcellular localization of the Arabidopsis fructokinases suggests that AtFRK6 is the only plastidic protein; the others are most likely cytosolic (Wolf Psort-http://www.genscript.com/wolf-psort.html, TargetP-http://www.cbs.dtu.dk/services/TargetP/) (Figure S1). This prediction is in line with mass spectrometry data showing that AtFRK1 and AtFRK7 are present in the cytosol (Ito et al., 2011), while AtFRK6 is present in the chloroplasts and, more specifically, in the stroma (Peltier et al., 2006; Rutschow et al., 2008; Zybailov et al., 2008; Ferro et al., 2010; Olinares et al., 2010; Helm et al., 2014).

In order to characterize the function of *AtFRK*s *in planta*, T-DNA mutants of five of the *AtFRK* genes (*AtFRK1,3,4,6,7*) were identified and obtained from ABRC stocks (https://abrc.osu.edu). The additional *AtFRKs* (i.e., *AtFRK2* and *AtFRK5*) did not have T-DNA mutants (in SALK, SAIL or WiscDsLox289 lines) or any other mutants in ABRC stocks. The AtFRK T-DNA Salk mutants will be referred as *frk1, frk3, frk4, frk6* and *frk7* (Table S1). The T-DNA Salk lines were characterized and we confirmed that they contained the T-DNA insertion in the corresponding genes based on PCR with primers from both sides of the T-DNA insertion (Table S2) and the absence of the related FRK mRNA (Figure S2).

### The arabidopsis double-mutant (*frk6 frk7*) exhibits a specific seed phenotype

**Figure 2:**
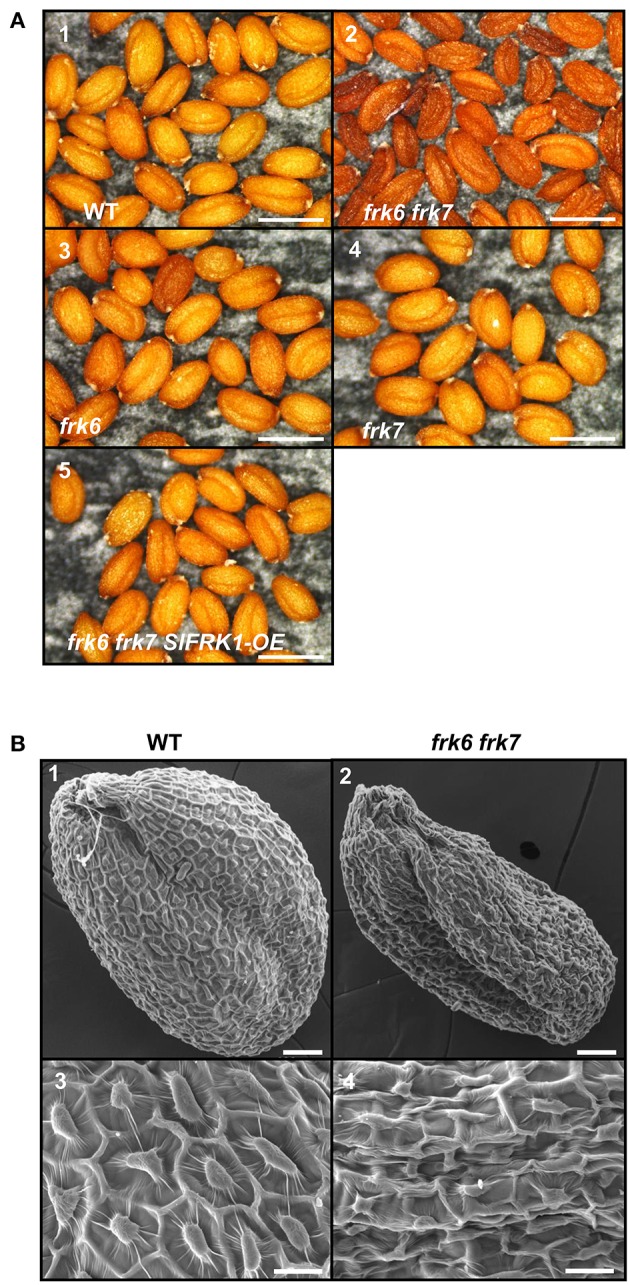
***frk6 frk7* double-mutant seed phenotypes. (A)** Dissecting microscope images of mature dry seed phenotypes. Seeds were harvested, placed on black paper and photographed under a dissecting microscope at maximal magnification. WT seeds (1), *frk6 frk7* double-mutant seeds (2), *frk6* seeds (3), *frk7* seeds (4), and T3 homozygous seeds of a cross between *frk6 frk7* and a line over-expressing *SlFRK1* (5). Bars–500 μm. **(B)** Scanning electron microscope micrographs of the different seeds. WT seed (1), *frk6 frk7* seed (2), WT seed surface (3), and *frk6 frk7* seed surface (4). Scale bars: (1–2)–50 μm; (3–4)–20 μm.

Neither one of the single T-DNA mutants of the Arabidopsis FRKs showed any visibly unusual phenotype when grown under normal growth conditions in soil or plates. Therefore, we made crosses between the individual lines to obtain homozygous double and multiple mutants, assuming that the AtFRKs' functions might be redundant. We obtained most of the double-mutant combinations and also created triple-, quadruple- and penta-mutants for different T-DNA lines. All of the double-mutants exhibited normal growth and produced normal seeds, except for a single double-mutant composed of the plastidic *AtFRK6* (*frk6*) and the cytosolic *AtFRK7* (*frk7*) whose seeds exhibited a unique phenotype. Those seeds were thin and dark and weighed 13% less than WT seeds (Figure 2A; Table 1). Scanning electron microscope images showed that seeds of *frk6 frk7* double-mutants were wrinkled at various degrees and had an abnormal surface (Figure 2B, Figure S3).

**Table 1:** **frk6 frk7 double mutant exhibit reduced seed weight**.

**Seed line**	**Average seed weight (μg)**
WT	20.4 ± 0.0
*frk7*	21.1 ± 0.6
*frk6*	20.3 ± 0.5
*frk6 frk7*	17.7 ± 0.4^*^

*Seeds were grown and harvested on the same time. More than 1000 seeds per plant (n = 3) were counted per sample and weighted for average seed weight. Asterisk denotes significant statistical difference from WT (p < 0.001)*.

### *frk6 frk7* double-mutant seedlings exhibit arrested growth that can be overcome by the addition of external sugar or the expression of tomato *SlFRK1*

**Figure 3:**
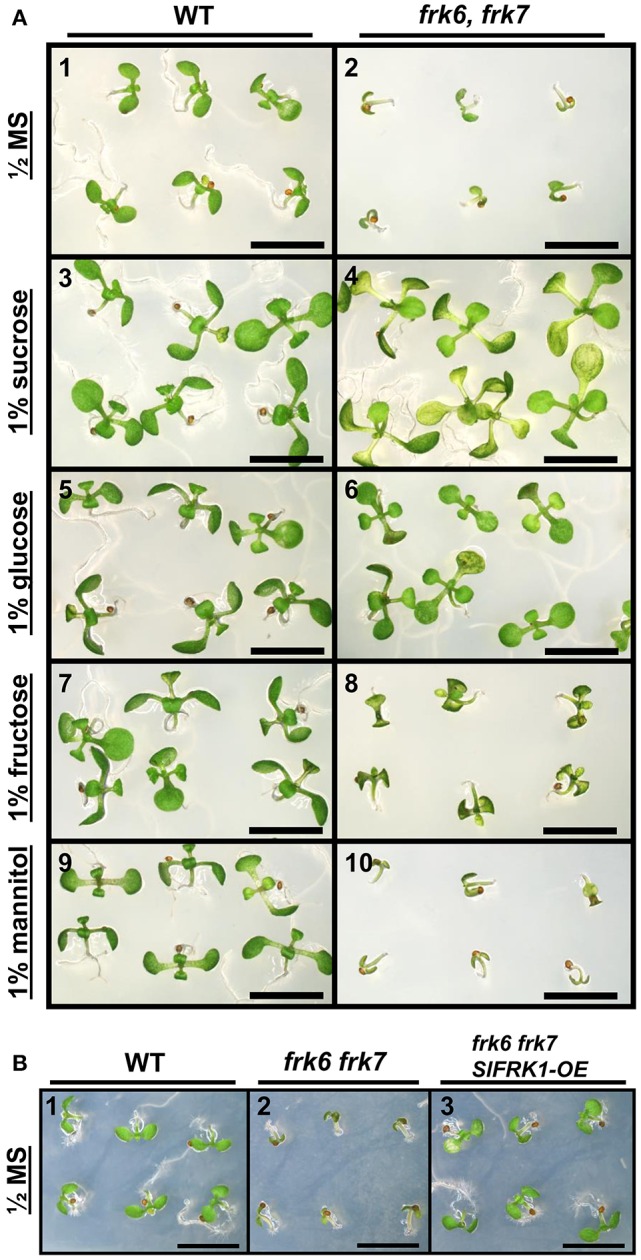
**Establishment of *AtFRK* double-mutant seedlings on artificial media. (A)** *frk6 frk7* seedlings show altered responses to different sugars. Seeds were sterilized and sown on half-strength MS media supplemented with different sugars. WT (1, 3, 5, 7, 9); *frk6 frk7* mutant (2, 4, 6, 8, 10); no sugar added (1–2), 1% sucrose (3–4), 1% glucose (5–6), 1% fructose (7–8) and 1% mannitol (9–10) added. Plates were photographed under a binocular microscope 10 days after they were transferred to the growth chamber. Scale bars–5 mm. **(B)** *SlFRK1* over-expression complements the *frk6 frk7* growth arrest. WT (1), *frk6 frk7* (2) and homozygous *frk6 frk7* over-expressing *SlFRK1* (3) seeds were sterilized and sown on half-strength MS media with no sugar added. Plates were photographed under a binocular microscope 10 days after they were transferred to the growth chamber. Scale bars–5 mm.

The abnormal shape and low weight of the double-mutant seeds raised the possibility that the germination and seedling development of these seeds might be affected. However, no growth defects were observed when the seeds of the double-mutant were sown in soil. When the seeds were grown on ½ MS plates with no added sugar, or with mannitol as control, the double-mutant seeds germinated, but stopped growing soon after germination (radicle and cotyledon emergence) and were unable to develop true leaves (Figures 3A1,2,9,10). The final germination percentage was not different from that of the WT seeds, but the germination rate was slightly slower (Figure S4). Neither of the other double-mutant combinations exhibited such seed germination and growth defects (not shown). The addition of 1% glucose or sucrose to the ½ MS plates overcame the growth arrest of the *frk6 frk7* double-mutant. This indicates that the growth arrest was a result of carbon starvation, perhaps due to insufficient accumulation of storage reserves (Figures 3A3–6). The addition of 1% fructose also allowed seedling establishment (Figures 3A7,8), but at a much slower rate than sucrose or glucose (Figures 3A4,6), suggesting that the double-mutant seedlings have a reduced ability to phosphorylate fructose. To test whether reduced fructokinase activity is indeed the primary cause of the seeds' wrinkled appearance and the arrested seedling growth, we crossed the double-mutant with an Arabidopsis line overexpressing the well-characterized tomato fructokinase 1 (*SlFRK1*) (Kanayama et al., 1997, 1998). Over-expression of *SlFRK1* against the background of the double-mutant restored both seed shape (Figure 2B) and seedling growth on ½ MS plates in the absence of sugar (Figure 3B), indicating that the double-mutant phenotype was indeed a result of a reduced ability to phosphorylate fructose.

### *frk6 frk7* seed phenotype does not stem from maternal effect

The phenotype of the *frk6 frk7* double-mutant seeds might be a result of altered carbohydrate production or transport from the mother plant to the seeds and/or seed-specific altered carbon metabolism. To determine whether the seed phenotype is due to maternal effect, we crossed the homozygote double-mutant *frk6 frk7* with pollen taken from WT, *frk6* homozygote or *frk7* homozygote plants (Table 2). The seeds of all of these crosses were normal with regular phenotypes (Table 2), indicating that the seed phenotype is not caused by reduced sugar production or reduced transport of sugar from the *frk6 frk7* homozygote mother plants to the seeds. The seed coats had the maternal genotype, which was *frk6 frk7* in all these crosses. However, despite that fact, the seeds were normal, indicating that the wrinkled phenotype is not a result of seed-coat effects.

**Table 2:** **Segregation of the phenotype of the progeny of the double-mutant crosses with WT, *frk7* and *frk6***.

**Cross (♀ × ♂)**	**Progeny**	**Expected genotypes (FRK6, FRK7)**			**Expected genotypic segregation of either endosperm or embryo**	**n**	**Distorted seeds**	**W seeds**
		seed coat	endosperm	embryo				
*frk6 frk7* × Col-0	f1	−/−, −/−	−−/+, −−/+	−/+, −/+	100%	39	0	39
*frk6 frk7* × *frk7*	f1	−/−, −/−	−−/+, −−/−	−/−, −/+	100%	41	0	41
*frk6 frk7* × *frk6*	f1	−/−, −/−	−−/−, −−/+	−/−, −/+	100%	29	0	29
*frk6 frk7* × *frk6*	f2	−/−, +/−	−−/−, −−/−	−/−, −/−	25%	324	83 (25.6%)	241 (74.4%)
		−/−, +/−	−−/−, −−/+	−/−, −/+	25%			
		−/−, +/−	−−/−, + +/−	−/−, +/−	25%			
		−/−, +/−	−−/−, + +/+	−/−, +/+	25%			

*-/- homozygous mutant allele, +/- heterozygous mutant allele, +/+ homozygous WT allele. Endosperm genotypes represented by three alleles as the endosperm is triploid with two identical maternal alleles and one paternal allele. The endosperm maternal alleles are also identical to the embryo maternal allele as they are a result of the megaspore haploid double mitosis. The genotype predicted to have distorted seeds is shaded in gray. n = number of seeds*.

We then examined the segregation of the phenotype among the F_2_ seeds of the cross between the double-mutant and the plastidic frk6 mutant (*frk6 frk7* × *frk6*). Twenty-five percent of these seeds exhibited the wrinkled double mutant phenotype (Table 2), indicating that this phenotype is caused by a seed-specific effect emanating from the endosperm or from the embryo itself.

### The double-mutant seeds show altered fatty acid metabolism

**Figure 4:**
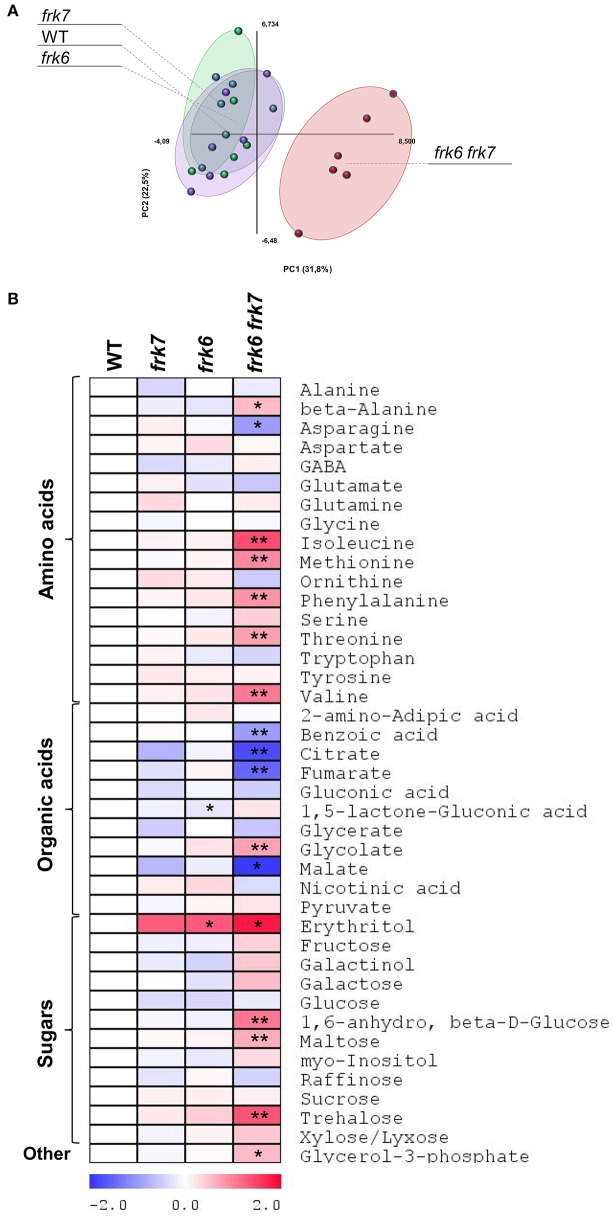
***frk6 frk7* double-mutant exhibits altered primary metabolism. (A)** PCA of primary metabolite levels. **(B)** Log2 values of the relative metabolic contents are presented as a heat map. Metabolic content was analyzed using GC-MS (*n* = 6). Significant differences between the mutant and the wild type (as determined using Student's *t*-test) are denoted by one asterisk (*P* < 0.05) or two asterisks (*P* < 0.01). Detailed results of the assay are presented in Supplemental File 1.

Transcriptomic data from microarray experiments suggest that the main *AtFRK*s expressed in developing seeds are *AtFRK1, AtFRK6* and *AtFRK7* (Schmid et al., 2005) (Figure S5). Expression data from different seed compartments during seed development showed that *AtFRK6* and *AtFRK7* are expressed in most seed compartments, with the highest levels of expression found in the embryo throughout the mid to late stages of its development (Le et al., 2010) (Figure S6). The mid to late embryo development stages are characterized by cell expansion and the accumulation of storage reserves, primarily oil. To examine the impact of *AtFRK6* and *AtFRK7* on carbohydrate metabolism and assess its importance for the accumulation of seed storage reserves, we used GC-MS to analyze the primary metabolites profiles of dry seeds of the *frk6 frk7* double-mutant, as well as of the single-mutants *frk6* and *frk7*. While there were only slight metabolite changes in each of the single-mutants, there was a major shift in metabolites in the double-mutant seeds (Figure 4A). The double-mutant seeds contained decreased levels of organic acids, such as citrate, fumarate, malate and benzoic acid, as well as increased levels of glycolate and glycerol 3-phosphate (Figure 4B). The decreased levels of TCA cycle organic acids (citrate, fumarate, and malate) suggest that the F6P that originates from fructose phosphorylated by AtFRK6 and AtFRK7 might be important for the glycolysis that feeds the TCA cycle and that glycolysis in the developing embryos of the double-mutant might be reduced.

**Figure 5:**
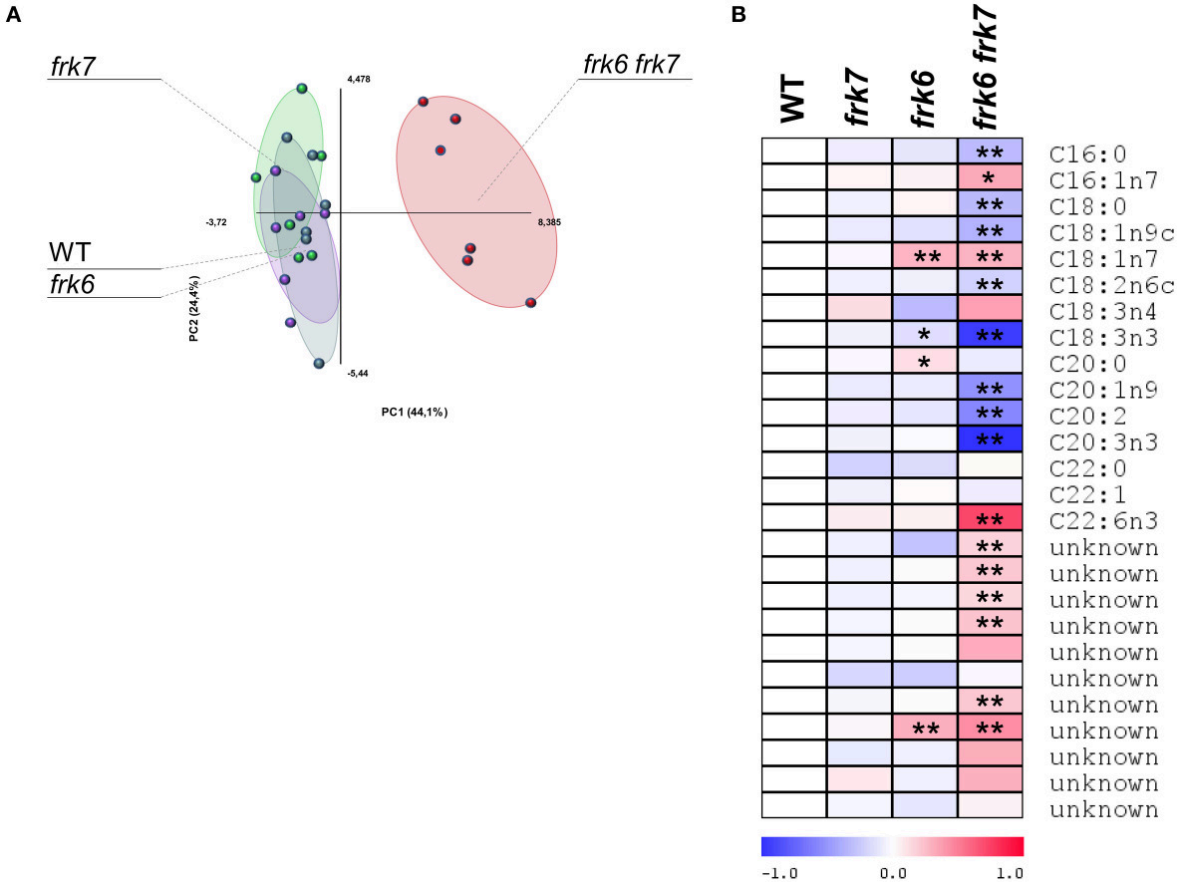
***frk6 frk7* double-mutant exhibits an altered lipid profile**. Twenty dry seeds per sample were used for lipid extraction. Lipid profiles were analyzed using GC-MS (*n* = 6). **(A)** PCA of the lipid profile. **(B)** Log2 values of the relative lipids are presented as a heat map. Significant differences between the mutant and the wild type (as determined using Student's *t*-test) are denoted by one asterisk (*P* < 0.05) or two asterisks (*P* < 0.01).

Glycolysis in maturing Arabidopsis seeds is highly important for plastidic acetyl-CoA formation for fatty acid metabolism and oil accumulation (Andre et al., 2007; Baud et al., 2007b). The wrinkled phenotype of the double-mutant seeds is similar to that of Arabidopsis WRINKLED1 mutants whose seeds accumulate less oil (Focks and Benning, 1998; Baud et al., 2007a). This similarity raises the possibility that the *frk6 frk7* double-mutant might have reduced fatty acid synthesis. To examine that possibility, we performed seed fatty acid profiling. The fatty acid profiles of *frk6* and *frk7* single-mutants were similar to that of WT (Figure 5A) with only small changes in *frk6* fatty acids. However, there was a major shift in the fatty acid profile of the *frk6 frk7* double-mutant (Figure 5A). The double-mutant showed significantly lower levels of many fatty acids, including palmitic acid (C16:0), stearic acid (C18:0), oleic acid (C18:1n9), linoleic acid (C18:2n6), alpha linolenic acid (C18:3n3), eicosenoic acid (C20:1n9), eicosadienoic acid (C20:2), and eicosatrienoic acid (C20:3n3), which were accompanied by increased levels of palmitoleic acid (C16:1n7), vaccenic acid (C18:1n7), docosahexaenoic acid (C22:6n3), and most of the unknown fatty acids (Figure 5B). The altered levels of fatty acids and, in particular, the decreases in C16:0, C18:0, and C18:1 suggests that fatty acid synthesis in plastids might be the main metabolic pathway affected by the combined loss of the cytosolic AtFRK6 and the plastidic AtFRK7.

**Figure 6:**
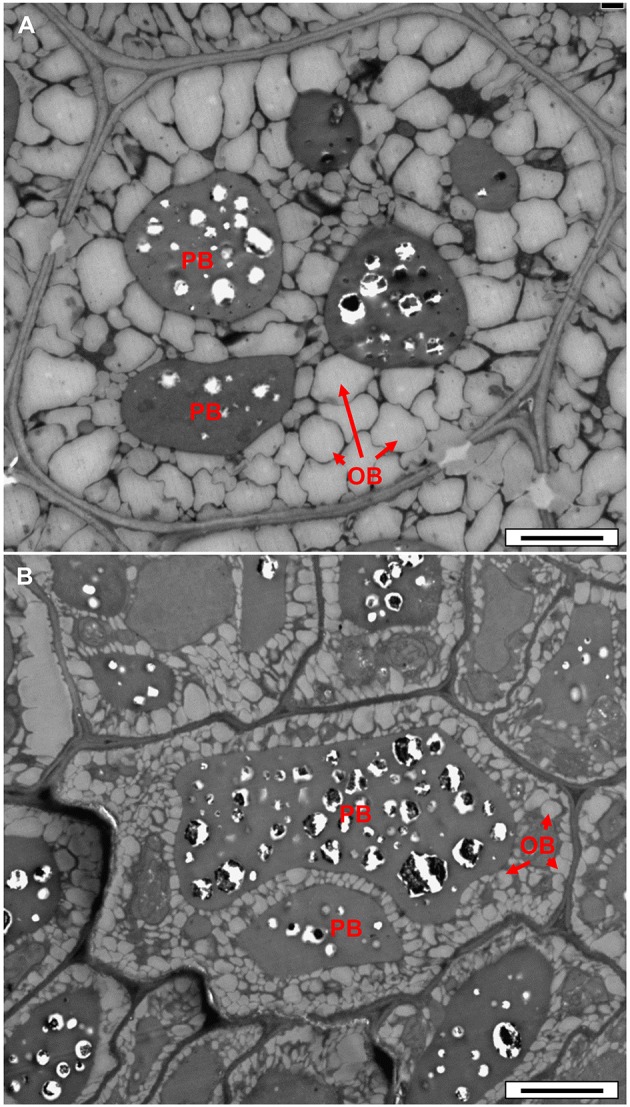
**Transmission electron microscope micrographs of dry seed embryonic cells. (A)** WT. **(B)** *frk6 frk7* mutant. OB, oil bodies; PB, protein bodies. Bars: 2 μm

In order to check whether reduced fatty acid synthesis in the double-mutant led to reduced oil accumulation, we examined dry seeds using transmission electron microscopy. A clear decrease in the area and volume of oil bodies (OB) was observed in the double-mutant embryo cells (Figure 6B) relative to the WT (Figure 6A), implying decreased oil content. No visual effect was observed in the single-cell layer of the endosperm of the double-mutant (Figure S7), suggesting that the *frk6 frk7* double mutations primarily affected the accumulation of storage reserves in the embryo.

### The arabidopsis quadruple-mutant (*frk1 frk4 frk6 frk7*) and penta-mutant (*frk1 frk3 frk4 frk6 frk7*) show altered vascular tissue development

**Figure 7:**
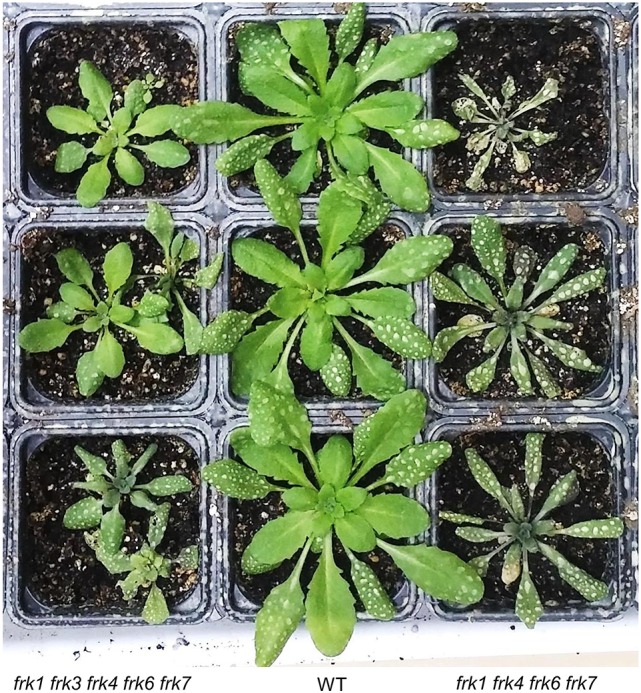
***AtFRK* quadruple- and penta-mutants exhibit leaf wilting after bolting**. WT, quadruple-mutant and penta-mutant Arabidopsis plants were grown in a growth chamber for 8 weeks under even-day conditions (12 h of light each day) and photographed when their leaves started to wilt. The white spots on the old leaves are residues from routine fungicide spraying.

Seeds of the quadruple- and penta-mutant plants that possessed both *frk6* and *frk7* mutations (*frk1 frk4 frk6 frk7* and *frk1 frk3 frk4 frk6 frk7*, respectively) were wrinkled even more than the *frk6 frk7* double-mutant (Figure S8) and stopped growing after germinating on ½ MS plates like *frk6 frk7* double-mutant. These lines grew normally in soil under long-day conditions (16/8 h, data not shown). However, when grown in soil under even-day (12/12 h) or short-day (8/16 h) conditions, the quadruple-mutant plants were smaller than the WT plants and the penta-mutant plants were even smaller, with rosette leaves that were half the size of those of the WT plants (Figure 7). The hypocotyls of both the mutants were shorter (2–3 mm) than those of the WT (4–5 mm) plants and, just before bolting, the mutants started wilting, dried out and died (Figure 7). The wilting phenotype points to lower water conductance, perhaps due to distorted vascular development as was previously shown in tomato *FRK* mutants (Damari-Weissler et al., 2009; Stein et al., 2016).

**Figure 8:**
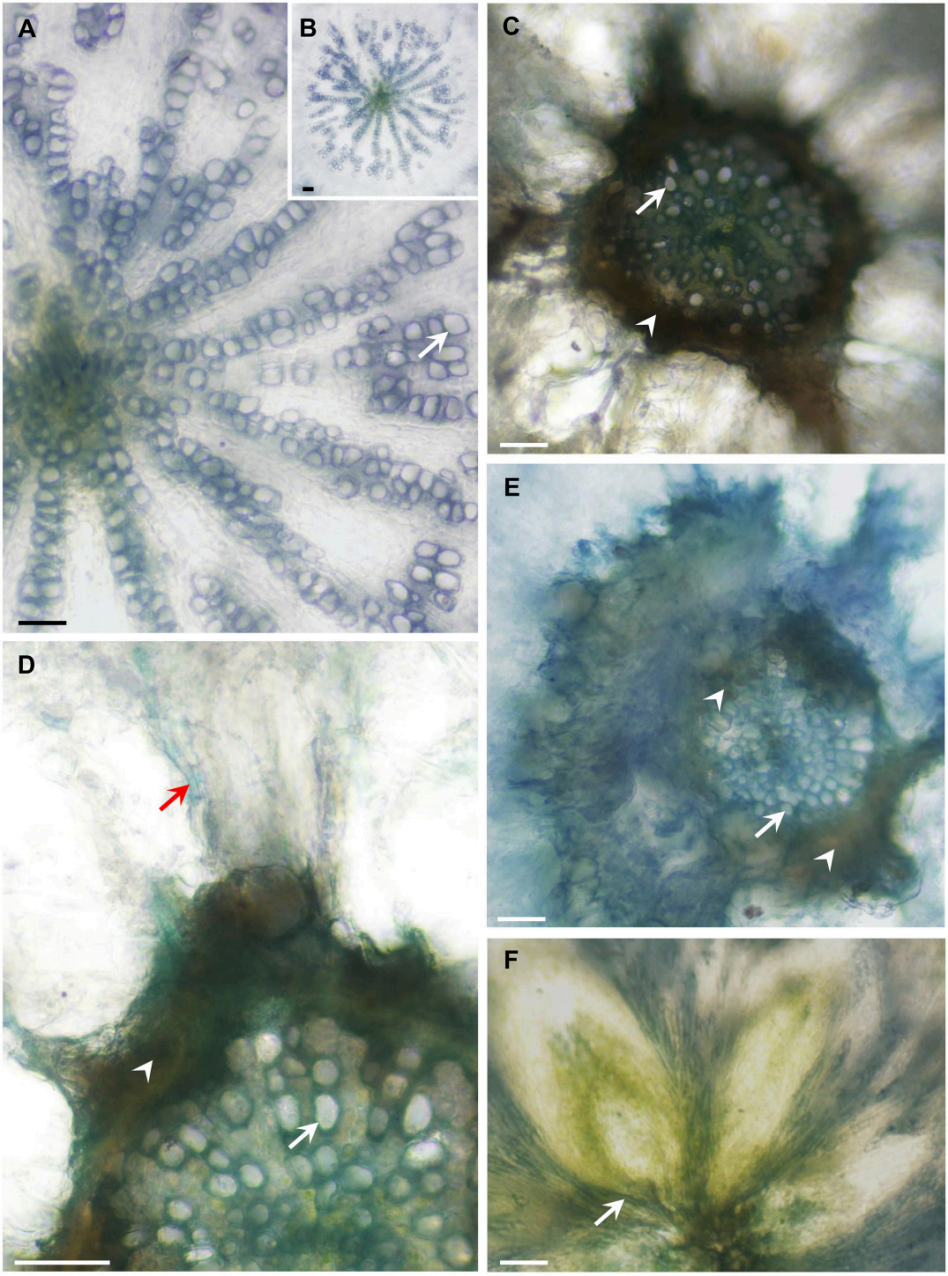
***AtFRK* quadruple- and penta-mutants exhibit altered vascular development**. The plants shown in Figure 9 were used for the anatomical analysis. Cross-sections of WT, quadruple- and penta-mutants of *Arabidopsis* were prepared and the appearance of the WT hypocotyls **(A,B)** was compared with that of the quadruple-mutant **(C,D,F)** and penta-mutant **(E)** hypocotyls, revealing a dark necrotic secretion (arrowheads) in the cambium that causes drastic secondary xylem reduction in both mutants **(C–E)**. **(A)** Wild-type hypocotyl showing about half of the vessels (arrows) differentiated in a ray pattern. **(B)** The entire xylem of the WT hypocotyl is shown in **(A)**. **(C)** Drastically reduced xylem area due to the limiting dark cambial necrotic secretion in the quadruple-mutant, which interrupted the typical WT radial vessel pattern **(A,B)**, demonstrating a sharp reduction in vessel number and vessel width. **(D)** Enlarged section of the quadruple-mutant with the same symptoms shown in **(C)**, which also shows a callose-loaded sieve tube (red arrow). **(E)** Massive necrotic secretion and limited xylem in the penta-mutant showing narrow vessels with thin secondary walls. **(F)** The vascular tissues at the base of the lower rosette leaves are free of necrotic secretions. Bars–100 μm.

To examine possible effects on vascular development in the Arabidopsis mutants, we analyzed the anatomy of these lines and found that the hypocotyls of the quadruple- and penta-mutants had limited xylem with narrow vessels that did not have a ray pattern; whereas WT plants had well-developed xylem (Figures 8A,B) with wide vessels that appeared in a regular ray pattern (Figure 8A). The mutants' secondary xylem was surrounded by cambium characterized by dark necrotic secretion (Figures 8C–E), which likely limited xylem growth and differentiation. Additionally, the sieve tubes of the mutants had high levels of callose along the hypocotyl (Figure 8D); whereas the sieve tubes of the WT were callose-free (not shown). Like the WT, both the leaves (Figure 8F) and the roots of the quadruple- and penta-mutants were free of any of the above symptoms. The heavy dark cambial necrotic secretion of the cambium and the reduced secondary xylem occurred in a limited region along the plant, only along the short mutant's hypocotyl (about 3 mm), creating a vascular bottleneck impeding the flow of water between the roots and the leaves.

## Discussion

### Arabidopsis FRK functions are highly redundant

As of today, the tomato FRKs are the best characterized FRKs with regard to biochemical characterization, intracellular localization and developmental functions. The fructose-phosphorylation activity of AtFRK6 (At1g66430) has been demonstrated previously (Arsova et al., 2010). Although the fructokinase activity of the other Arabidopsis FRKs has not been directly demonstrated, there are several lines of evidence to suggest that they are indeed fructose-phosphorylating enzymes. The complementation of the double-mutant phenotype by glucose or sucrose and only partially by fructose indicates reduced **fructose-specific** phosphorylation activity. The double-mutant seeds (*frk6 frk7)* accumulated 30% more fructose than the *frk6* mutant alone, suggesting that AtFRK7 is also a fructose-phosphorylating enzyme. The complementation of the *frk6 frk7* double-mutant by the well-characterized tomato FRK1, which has confirmed fructose-phosphorylation activity and has high identity to Arabidopsis FRK7 (Figure 1) further supports the fructose-phosphorylation activity of AtFRK7. Lastly, the fructose-binding domain composed of eight amino acids of well-characterized FRKs (Chua et al., 2010) is 100% conserved across all seven Arabidopsis FRKs and four tomato FRKs (five of which have proven fructose phosphorylation activity). We believe that this evidence substantially supports the assumption that these genes encode fructose-phosphorylating enzymes.

The major tomato cytosolic fructokinase, *SlFRK2*, is necessary for vascular development and suppression of *SlFRK2* together with the plastidic *SlFRK3* has an even more severe effect on vascular development. Unlike in tomato, neither of the single Arabidopsis *FRK* mutant lines showed any phenotype suggesting a high degree of redundancy of *AtFRK* functions. The seed-specific phenotype was observed only in the *frk6 frk7* double-mutant; whereas growth inhibition, leaf wilting and distortion of vascular development where found only in the quadruple- and penta-mutants. This might not be surprising considering that the entire Arabidopsis genome has undergone genome duplication followed by large rearrangements (Blanc et al., 2000). An example of this redundancy can be observed in the *AtFRK2* and *AtFRK3* genes, which are located side by side on chromosome 1 and share over 85% identity in both amino acid and mRNA sequences. Those probable redundant genes are likely a result of a tandem duplication event that occurred after genome duplication.

### *AtFRKs* play a key role in carbon metabolism and the accumulation of storage reserves in developing seeds

The *frk6 frk7* seed phenotype highly resembles that of the seed-filling WRINKLED1 mutants. While early stages of seed development are correlated with cell divisions and embryo morphogenesis, the later stages are correlated with cell expansion and the accumulation of storage reserves, primarily oil. The spatial and temporal expression patterns of *AtFRK6* and *AtFRK7* (Figures S5, S6) suggest that they might play a role in carbon metabolism in embryos during seed maturation and the accumulation of storage reserves. Maturation and storage-reserve accumulation in seeds of *Brassica napus*, an oil-accumulating, close relative species, is characterized by high sucrose to hexose ratios in the liquid endosperm, which suggest that sucrose is probably the main carbon source entering the embryos (Schwender and Ohlrogge, 2002; Hill et al., 2003). Similarly, developing embryos of the triple mutant of *SWEET11,12,15* sucrose transporters of Arabidopsis exhibit defective sucrose uptake and that mutant also exhibits a wrinkled phenotype and reduced seed weight (Chen et al., 2015). Sucrose is probably cleaved within the seeds by sucrose synthase (SUS) as SUS activity in *B. napus* seeds is much higher than alkaline invertase activity (Hill et al., 2003). The expression pattern of Arabidopsis *AtSUS2* and *AtSUS3* also suggest that SUS plays a key role in Arabidopsis storage-reserve accumulation (Fallahi et al., 2008). SUS cleaves sucrose into fructose and UDP-Glc and, accordingly, it is expected that FRK activity might be important for fructose phosphorylation. In fact, high levels of FRK activity have been observed in developing embryos of *B. napus* (Hill et al., 2003) and the maturing embryos of WRINKLED1 Arabidopsis mutants show almost no FRK activity (Baud and Graham, 2006), supporting the importance of FRKs for embryo carbon metabolism and storage-reserve accumulation. Accordingly, metabolite profiling of the *frk6 frk7* double-mutant seeds revealed altered levels of organic acids, sugars and amino acids, indicating significant changes in carbon metabolism (Figure 4). Furthermore, the decrease in the TCA-cycle organic acids (citrate, fumarate and malate) points to reduced glycolysis and implies that F6P that originated from the phosphorylation of fructose by AtFRK6 and AtFRK7 is a primary substrate that fuels seed glycolysis and the accumulation of storage reserves.

The double-mutant *frk6 frk7* seeds failed to grow after germinating on ½ MS plates unless supplemented with sucrose or glucose, supporting the notion that these seeds lack sufficient storage compounds for seedling establishment. Seedling establishment did occur in soil and we assume that organic matter in the soil complemented the lack of storage reserves in the seeds. The complementation of seedling establishment by sucrose on ½ MS plates is similar to that observed for WRINKLED1 mutants. However, WRINKLED1 mutants also exhibit reduced germination rates and reduced establishment in soil (Cernac et al., 2006), perhaps due to the fact that WRINKLED1 is a transcription factor that regulates the expression of many carbohydrate-metabolism genes (Focks and Benning, 1998; Ruuska et al., 2002; Baud et al., 2007a). The addition of fructose to the growth medium improved the growth of WT seedlings, but had almost no effect on *frk6 frk7* mutants, indicating that these FRKs may function through seedling establishment as well.

### FRKs rather than HXKs are central for seed development

**Figure 9:**
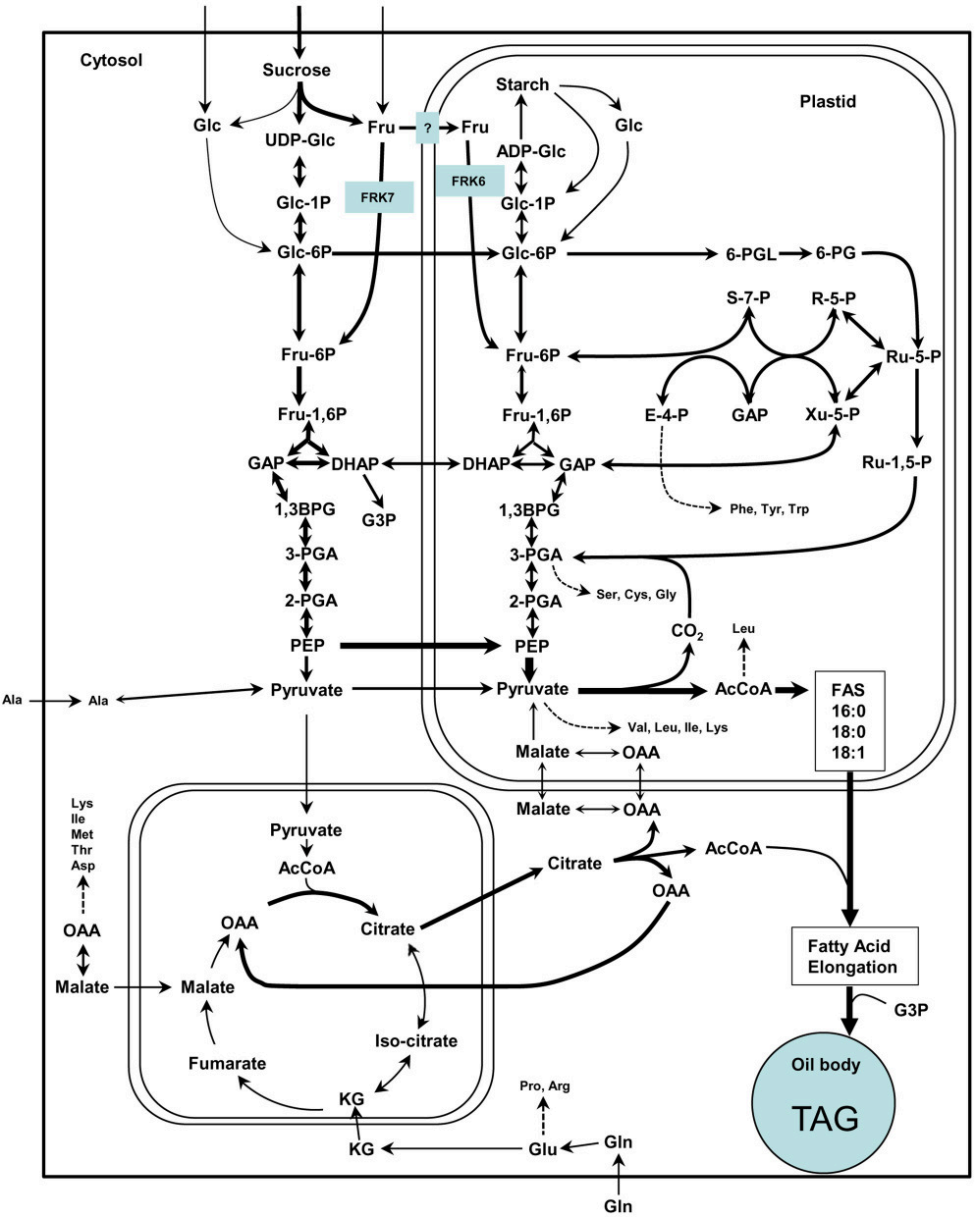
**Simplified scheme of the suggested model for cytosolic and plastidic FRK for glycolysis and fatty acid synthesis in Arabidopsis**. This scheme is adapted from Baud et al. (2008) in which arrow thickness are proportional to net carbon fluxes based on biochemical data and transcriptional profiling of maturing seeds (White et al., 2000; Schwender et al., 2004, 2006). ADP-Glc, adenosine diphosphoglucose; AcCoA, acetyl-coenzyme A; 1,3-BPG, 1,3-bisphosphoglycerate; DHAP, dihydroxyacetone-3-phosphate; E-4-P, erythrose-4-phosphate; Fru, fructose; Fru-1,6-P, fructose-1,6-bisphosphate; Fru-6-P, fructose-6-phosphate; GAP, glyceraldehyde-3-phosphate; Glc, glucose; Glc-1-P, glucose-1-phosphate; Glc-6-P, glucose-6-phosphate; KG, alpha-ketoglutarate; OAA, oxaloacetate; 6-PG, 6-phosphogluconate; 6-PGL, 6-phosphogluconolactone; PEP, phosphoenolpyruvate; 2-PGA, 2-phosphoglycerate; 3-PGA, 3-phosphoglycerate; R-5-P, ribose-5-phosphate; Ru-1,5-P, ribulose-1,5-bisphosphate; Ru-5-P, ribulose-5-phosphate; S-7-P, sedoheptulose-7-phosphate; UDP-Glc, uridine diphosphoglucose; Xu-5-P, xylulose-5-phosphate.

Glycolysis in maturing Arabidopsis seeds is highly important for fatty acid synthesis and oil accumulation (Andre et al., 2007; Baud et al., 2007b). Oil accumulation in Arabidopsis seeds is primarily dependent on *de novo* fatty acid synthesis in plastids, for which acetyl-CoA from pyruvate generated by the glycolytic pathway is a raw material (Figure 9). A similar wrinkled seed phenotype with reduced fatty acid synthesis was observed among mutants for the last plastidial glycolytic enzyme, the plastidic pyruvate kinase, which is responsible for converting phosphoenol pyruvate (PEP) into pyruvate in plastids. That pyruvate is the main source of acetyl-CoA for *de novo* fatty acid synthesis (Andre et al., 2007; Baud et al., 2007b). The contribution of HXKs to acetyl-CoA for fatty acid synthesis is less clear as cytoplasmic HXKs are associated with the mitochondria, together with other glycolytic enzymes, and presumably supply pyruvate to the mitochondrial TCA cycle (Giegé et al., 2003). Furthermore, in developing seeds, the expression levels of the cytoplasmic HXKs, *AtHXK1* and *AtHXK2*, are much lower than those of FRKs (Figure S9) and unlike the *frk6 frk7* double-mutant, the double-mutant of the major cytosolic *AtHXK1* and the single plastidic *AtHXK3* (*gin2 hxk3*) did not exhibit any abnormalities in seed shape or development (Figure S10). These results suggest that FRKs rather than HXKs are the primary gates for the synthesis of fatty acid and the accumulation of storage reserves in developing Arabidopsis seeds.

### *frk6 frk7* double-mutant exhibits reduced fatty acid synthesis

Fatty-acid profiling of the single *frk6* and *frk7* mutants showed that the cytosolic and the plastidic *AtFRKs* alone have little effect on fatty acid composition. Yet, in the double-mutant, there was a decrease in all of the major fatty acids found in the wild type, including the most abundant linoleic acid (C18:2n6), alpha linolenic acid (C18:3n3) and eicosenoic acid (C20:1n9), as well as the primary fatty acids that are formed within the plastids, palmitic acid (C16:0), stearic acid (C18:0) and oleic acid (C18:1n9), indicating reduced fatty acid synthesis in the double mutant. The significantly smaller oil bodies in *frk6 frk7* dry seeds also support the importance of *AtFRK6* and *AtFRK7* for fatty acid synthesis and oil accumulation. Most of the oil in Arabidopsis seeds accumulates in the embryos during seed maturation (Li et al., 2006) and the spatial and temporal expression patterns of *AtFRK6* and *AtFRK7* (Figures S5, S6) further support the notion that these two enzymes play a central role in fatty acid synthesis. The observed increase in some fatty acids, such as palmitoleic acid (C16:1n7) and vaccenic acid (C18:1n7), in the double-mutant is probably limited to the endosperm or seed coat as both of these fatty acids are found at very low levels in the embryos (Penfield et al., 2004; Li et al., 2006).

Changes in the amount of other metabolites in *frk6 frk7* seeds further support the hypothesized reduced fatty acid synthesis. The increase in glycerol-3-phosphate (G3P), the backbone of triacyl glycerol (TAG = oil), indicates a low availability of fatty acids for TAG assembly. The accumulation of certain amino acids (i.e., beta-alanine, isoleucine, methionine, threonine and valine) might also be a result of reduced fatty acid synthesis, which would allow more carbon to be directed toward amino acid synthesis.

Another interesting altered metabolite is erythritol. Erythritol was the only metabolite to be altered in the single mutants as well. Erythritol levels were 2.5-fold higher in both single mutants (although not statistically significant in *frk7* mutant) and 3.5-fold higher in the double-mutant, indicating an additive effect. We assume that the increase in erythritol might be the result of more glucose-6-phosphate (G6P) entering the oxidative pentose phosphate pathway (OPPP). The entrance of G6P to the OPPP yields erythrose-4P and F6P (Figure 9) in which the latter may compensate for the reduced fructose phosphorylation. Erythrose-4P may then be used for erythritol synthesis or enter the shikimate pathway. The increase in the amounts of erythritol and phenyl alanine produced by the shikimate pathway in plastids may support this speculation.

### *AtFRKs* are important for both cytosolic and plastidic glycolysis in arabidopsis embryos

Previously collected transcriptomic data suggest that the cytosolic glycolysis is the main glycolytic pathway in developing seeds for the production of PEP (White et al., 2000; Ruuska et al., 2002), which is then imported into plastids by PPT before its subsequent conversion to pyruvate (Kubis et al., 2004). PEP is imported by plastids isolated from embryos utilized for fatty acid synthesis at rates that are sufficient to account for one-third of the rate of fatty acid synthesis in *B. napus* (Kubis et al., 2004). It has also been shown that the enzymes upstream of the plastidic pyruvate kinase in the glycolytic pathway are not essential for fatty acid synthesis as mutations in both plastidic enolase and plastidic glycerate mutase had no effect on oil accumulation, indicating that cytosolic glycolysis is sufficient to support the carbon flux required for oil production in developing embryos (Andriotis et al., 2010). Our results are in line with these observations showing that the plastidic FRK6 is not essential for fatty acid synthesis. However, we also show that the cytosolic FRK7 is also not essential for fatty acid synthesis. Only the double-mutant exhibited reduced fatty acid synthesis, suggesting that FRK6 and FRK7 are likely key enzymes controlling the flux of carbon to plastidic and cytosolic glycolysis, respectively, and together are essential for fatty-acid synthesis.

### Arabidopsis FRKs are important for vascular tissue development and function

It appears that in addition to their role in seed development, *AtFRK*s are also important for vascular development. Nevertheless, the seed phenotype resulted from a specific lack of FRK6 and FRK7 expression in the seeds.

Both the quadruple- and penta- Arabidopsis FRK mutants had necrotic secretions around the cambium which cause reduced cambial activity, xylem formation and differentiation. It appears that the growth of the penta-mutant is more inhibited than that of the quadruple-mutant (Figure 7), possibly due to the effects of the additional *frk3* mutation on cambium activity and xylem formation. Yet, the wilting phenotype of the quadruple-mutant appeared slightly earlier than the similar phenotype appeared in the penta-mutant (Figure 7). This may be due to the bigger leaves and rosettes of the quadruple-mutant, which probably enhance water loss and wilting. Nevertheless, the end result was similar for both the quadruple- and the penta-mutants: desiccation and death.

The anatomical characteristics and the wilting phenotypes of the quadruple- and penta-mutants were similar to those observed in tomato plants in which cytosolic *FRK2* and plastidic *FRK3* were repressed (Stein et al., 2016). It appears that in both tomato and Arabidopsis, the observed vascular phenotype can be attributed to the suppression of the plastidic FRK and one or more of the cytosolic FRKs.

Expression data and proteomic analyses support the importance of *AtFRK*s for vascular development showing expression of *AtFRK1,3,4,6,7* in vascular tissues, but these FRKs may have overlapping roles in which they complement each other. Yet, in both seeds and vascular tissues, *AtFRK*s may contribute to the strength of the cell walls, as deformed cell walls that cause abnormal embryo cell shapes are observed in the wrinkled seeds of the double mutant and the vascular tissues of the multiple mutants (Figures 6, 8).

The results of this work suggest that F6P generated by FRK activity might be an important carbon source for cytosolic and plastidic glycolysis in developing Arabidopsis embryos and that, in Arabidopsis, FRKs are also important for vascular tissue development. However, we predict that additional components are probably also required to determine the fate of F6P, that is, whether it is directed to fatty acid synthesis or to other metabolic pathways.

## Author contributions

OS and DG planned and designed the research and wrote the manuscript. OS, TA, IK, HZ, VB, OD, and RA performed the experiments. OS, TA, IK, RA, AF, and DG analyzed the data.

## Funding

This research was supported by grant IS-4541-12 from BARD, the United States–Israel Binational Agricultural and Development Fund.

### Conflict of interest statement

The authors declare that the research was conducted in the absence of any commercial or financial relationships that could be construed as a potential conflict of interest.

## Abbreviations

FRK: Fructokinase
SUS: Sucrose synthase
HXK: Hexokinase
TCA: Tricarboxylic acid
WT: Wild type
TAG: Triacyl glycerol
G6P: Glucose-6-phosphate
F6P: Fructose-6-phosphate
PEP: Phosphoenol pyruvate
G3P: Glycerol-3-phosphate
UDP: Uridine diphosphate.
