# Corrigendum: Arabidopsis Fructokinases Are Important for Seed Oil Accumulation and Vascular Development

**DOI:** 10.3389/fpls.2017.00303

**Published:** 2017-03-06

**Authors:** Ofer Stein, Tamar Avin-Wittenberg, Ina Krahnert, Hanita Zemach, Vlada Bogol, Oksana Daron, Roni Aloni, Alisdair R. Fernie, David Granot

**Affiliations:** ^1^Volcani Center, Institute of Plant Sciences, Agricultural Research OrganizationBeit Dagan, Israel; ^2^Robert H. Smith Faculty of Agriculture, Institute of Plant Sciences and Genetics in Agriculture, Food and Environment, Hebrew University of JerusalemRehovot, Israel; ^3^Max-Planck-Institut für Molekulare PflanzenphysiologiePotsdam-Golm, Germany; ^4^Department of Plant and Environmental Sciences, Hebrew University of JerusalemGivat Ram, Jerusalem, Israel; ^5^Department of Life Sciences, Ben-Gurion UniversityBeer-Sheva, Israel; ^6^Department of Plant Sciences, Tel Aviv UniversityTel Aviv, Israel

**Keywords:** fructokinase, seed oil, xylem, fatty acid, carbon metabolism

## Error in Figure 1

In the original article, there was a mistake in Figure 1 as published. The gene ID for AtFRK3 and AtFRK4 that were used in the mega4 software for creating the phylogenetic tree were incorrect. AtFRK3 (At3g59480) should read AtFRK3 (At1g06020) and AtFRK4 (At4g06030) should read AtFRK4 (At3g59480) as in the text, Table S1 and uniprot annotation for arabidopsis probable fructokinases. The corrected Figure 1 appears below. The authors apologize for this error and state that this does not change the scientific conclusions of the article in any way.

**Figure 1:**
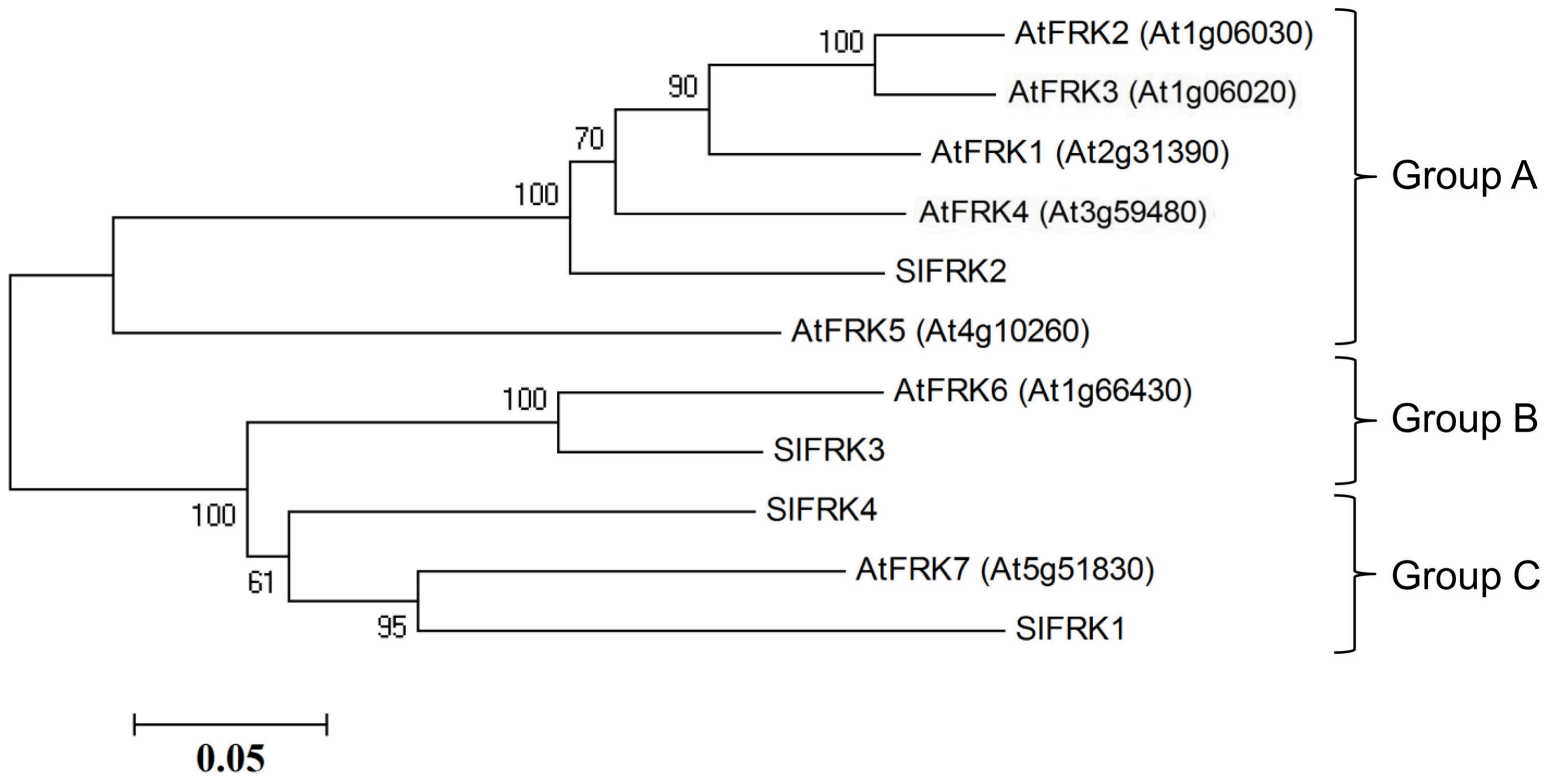
**Phylogenetic relationships between tomato and Arabidopsis fructokinases**. Phylogenetic evolutionary analysis was conducted using *MEGA* version 4 (Tamura et al., 2007). This phylogenetic tree is based on the following protein sequences: SlFRK1 (NP_001233893), SlFRK2 (NP_001233888), SlFRK3 (NP_001234396), SlFRK4 (NP_001234206), At5g51830 (NP_199996), At1g66430 (NP_564875), At4g10260 (NP_192764), At3g59480 (NP_191507), At1g06030 (NP_172093), At1g06020 (NP_172092), and At2g31390 (NP_180697).

### Conflict of interest statement

The authors declare that the research was conducted in the absence of any commercial or financial relationships that could be construed as a potential conflict of interest.
