# Supplementary material for: Arabidopsis Fructokinases Are Important for Seed Oil Accumulation and Vascular Development
Source: Front Plant Sci. 2017 Jan 10;7:2047. doi: 10.3389/fpls.2016.02047 (PMC5222831; doi:10.3389/fpls.2016.02047)
Supplement: Supplementary file 1 [file Presentation1.PDF]

Figure S1

A

test*k used for kNN is: 14*	
At5g51830	cyto: 8
At1g06030	cyto: 9.5
At1g06020	cyto: 9
At3g59480	cyto: 9
At4g10260	cyto: 7
At1g66430	chlo: 14
At2g31390	cyto: 7.5

B

```
### targetp v1.1 prediction results ##################################
Number of query sequences:  7
Cleavage site predictions not included.
Using PLANT networks.

Name                  Len     cTP    mTP     SP  other  Loc  RC
----------------------------------------------------------------------
At5g51830             343   0.111  0.046  0.104  0.935   _    1
At1g06030             329   0.150  0.056  0.099  0.754   _    2
At1g06020             345   0.180  0.044  0.165  0.643   _    3
At3g59480             326   0.039  0.037  0.477  0.608   _    5
At4g10260             324   0.147  0.045  0.383  0.500   _    5
At1g66430             384   0.975  0.109  0.002  0.020   C    1
At2g31390             325   0.055  0.049  0.297  0.635   _    4
----------------------------------------------------------------------
cutoff                      0.000  0.000  0.000  0.000
```

Figure S2

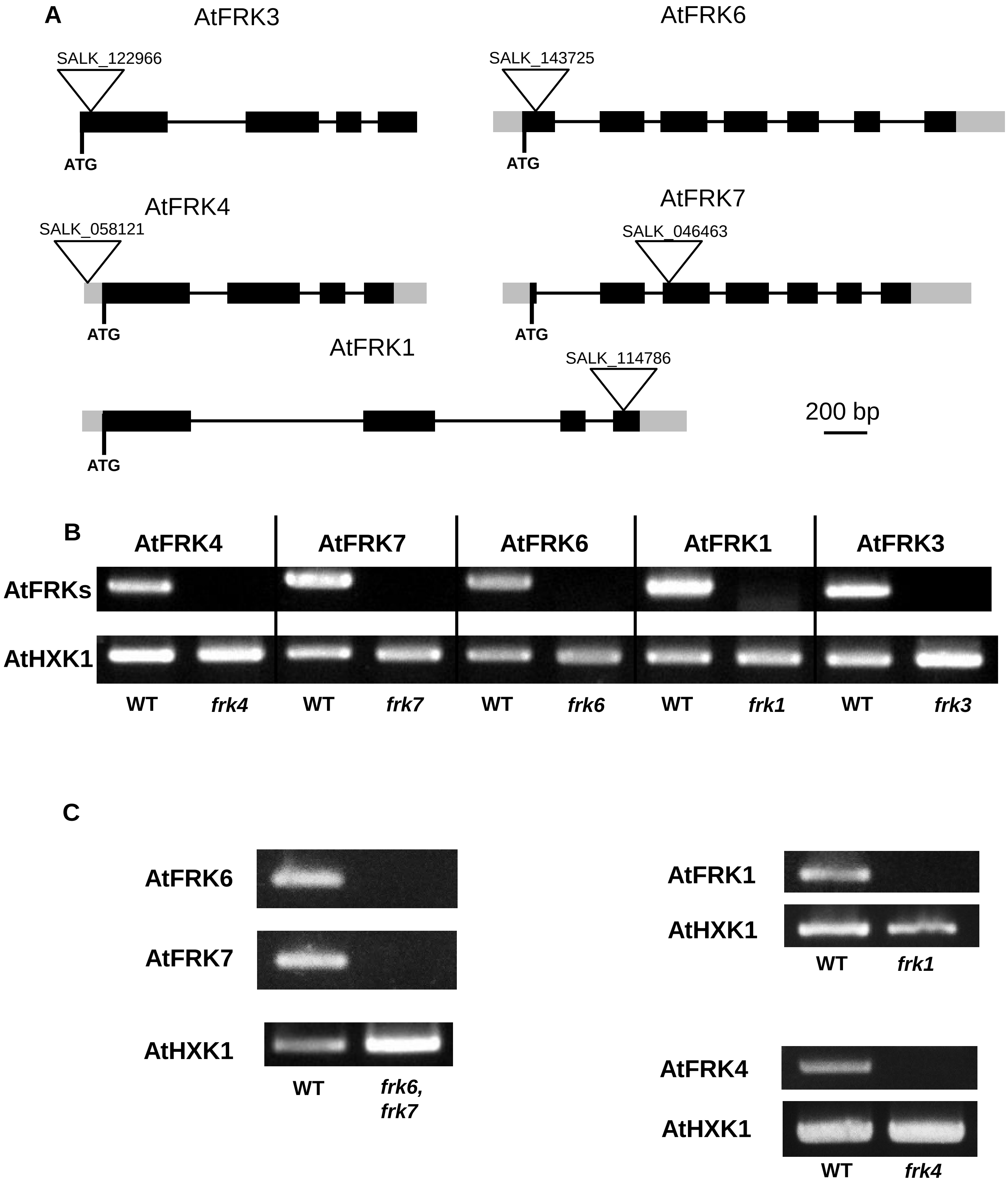

A
AtFRK3
SALK_122966
ATG
AtFRK6
SALK_143725
ATG
AtFRK4
SALK_058121
ATG
AtFRK7
SALK_046463
ATG
AtFRK1
SALK_114786
ATG
200 bp
B
AtFRK4
AtFRK7
AtFRK6
AtFRK1
AtFRK3
AtFRKs
AtHXK1
WT
frk4
WT
frk7
WT
frk6
WT
frk1
WT
frk3
C
AtFRK6
AtFRK7
AtHXK1
WT
frk6, frk7
AtFRK1
AtHXK1
WT
frk1
AtFRK4
AtHXK1
WT
frk4

Figure S3

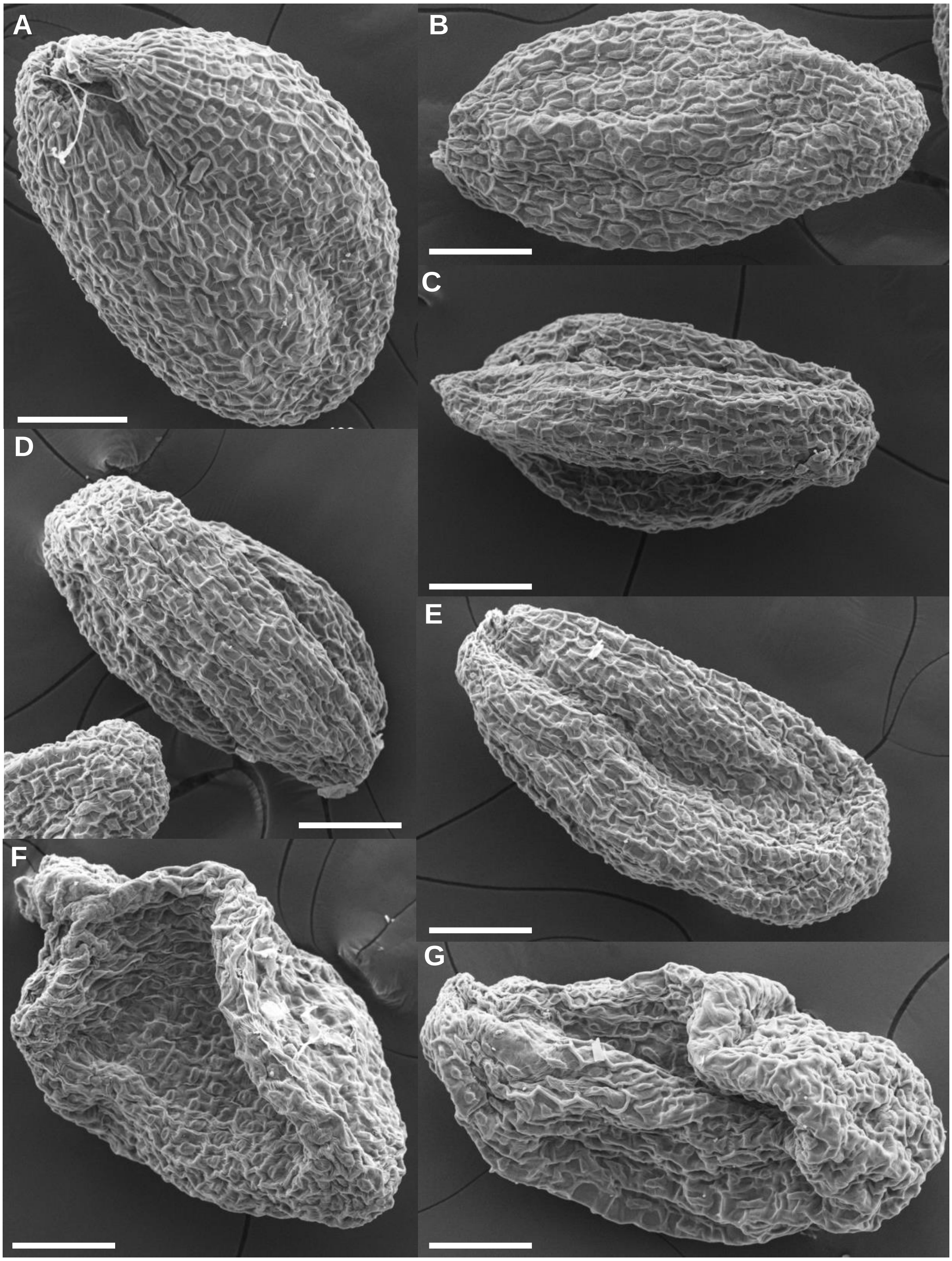

Figure S4

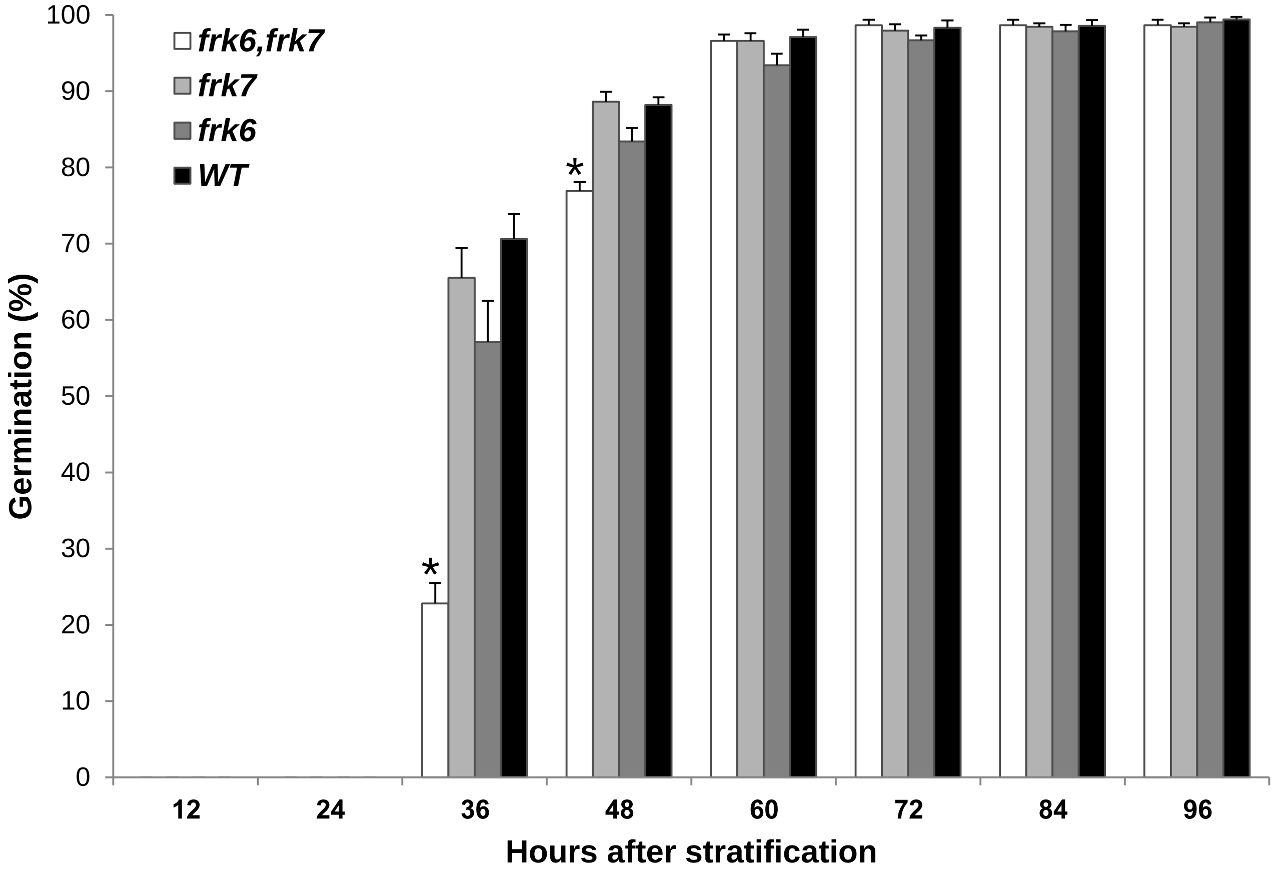

frk6,frk7
frk7
frk6
WT
Germination (%)
Hours after stratification
0
10
20
30
40
50
60
70
80
90
100
12
24
36
48
60
72
84
96
*
*

Figure S5

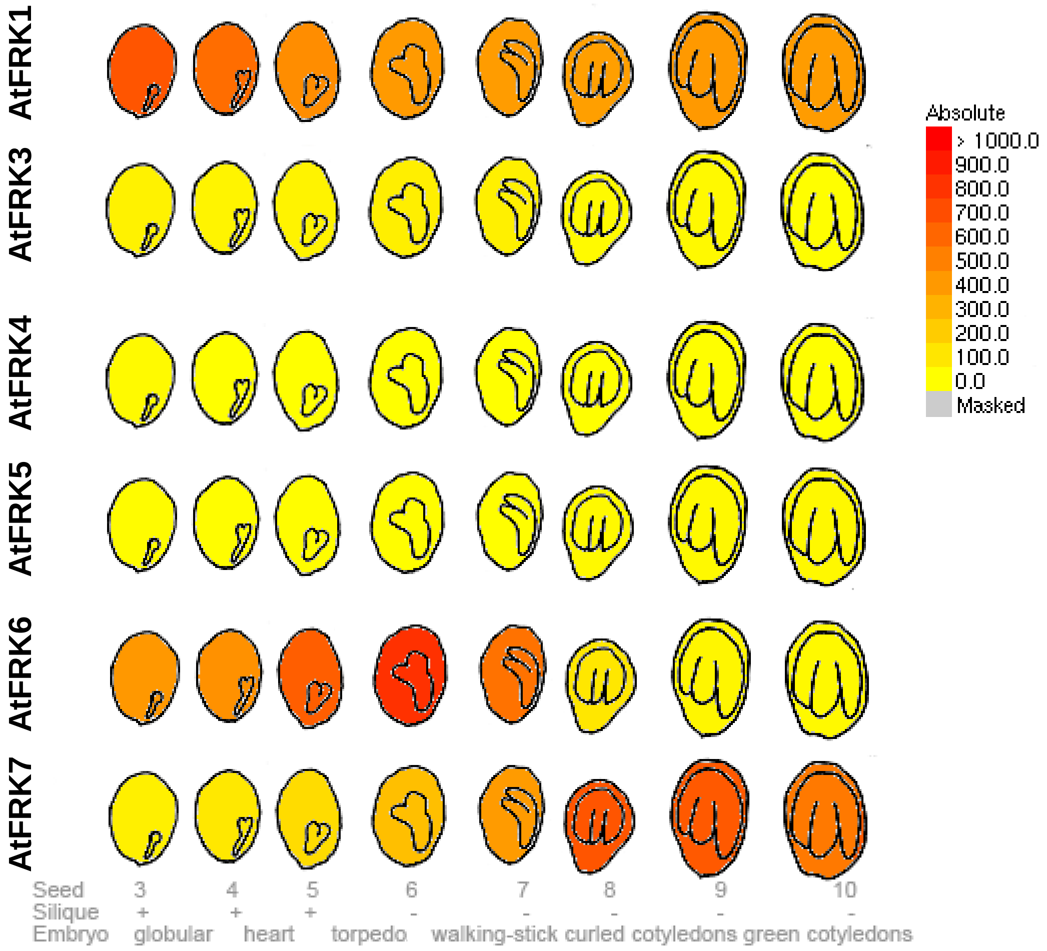

AtFRK1
AtFRK3
AtFRK4
AtFRK5
AtFRK6
AtFRK7
Absolute
> 1000.0
900.0
800.0
700.0
600.0
500.0
400.0
300.0
200.0
100.0
0.0
Masked
Seed 3 4 5 6 7 8 9 10
Silique + + + - - - - -
Embryo globular heart torpedo walking-stick curled cotyledons green cotyledons

Figure S6

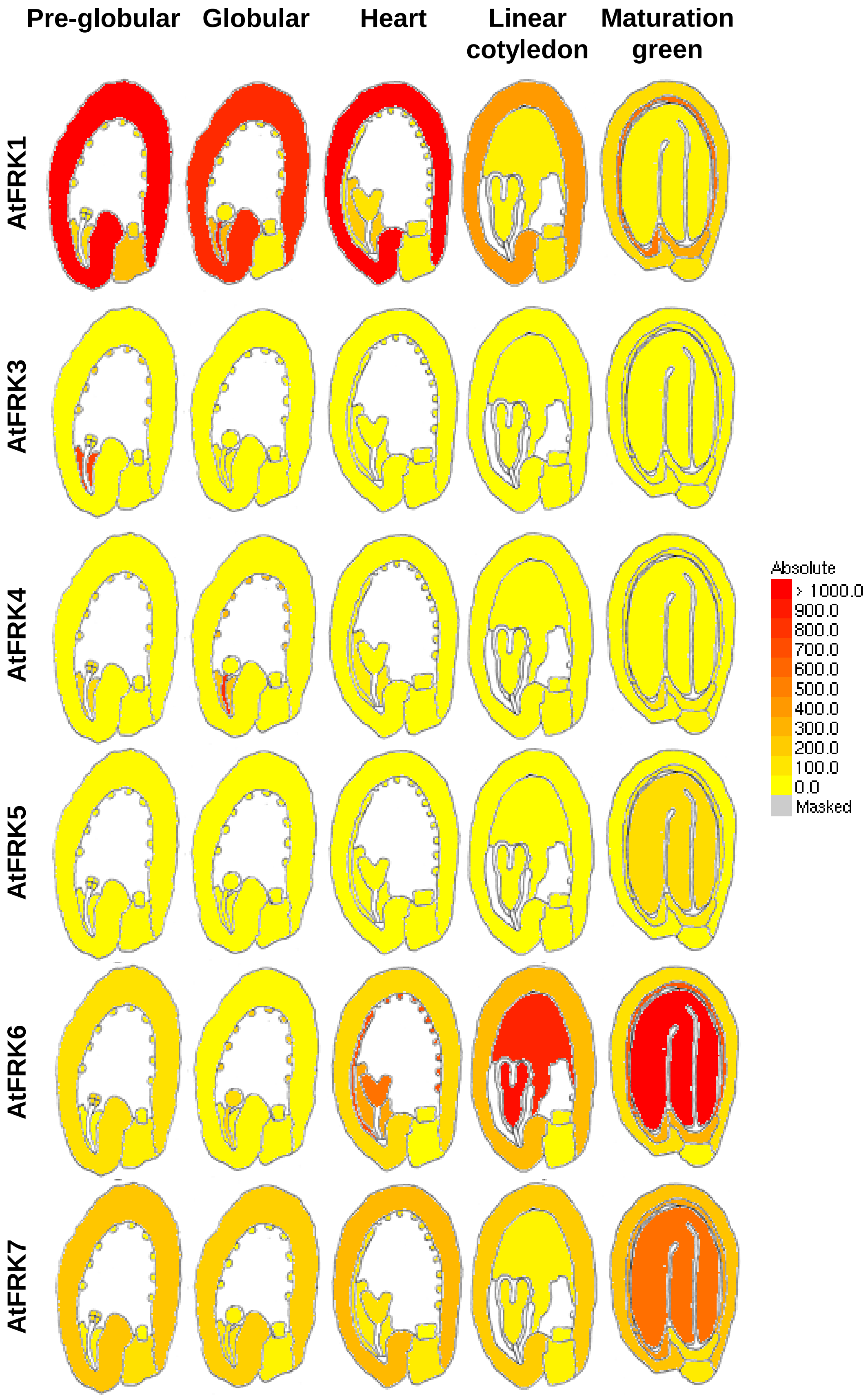
Pre-globular
Globular
Heart
Linear cotyledon
Maturation green
AtFRK1
AtFRK3
AtFRK4
AtFRK5
AtFRK6
AtFRK7
Absolute
> 1000.0
900.0
800.0
700.0
600.0
500.0
400.0
300.0
200.0
100.0
0.0
Masked

Figure S7

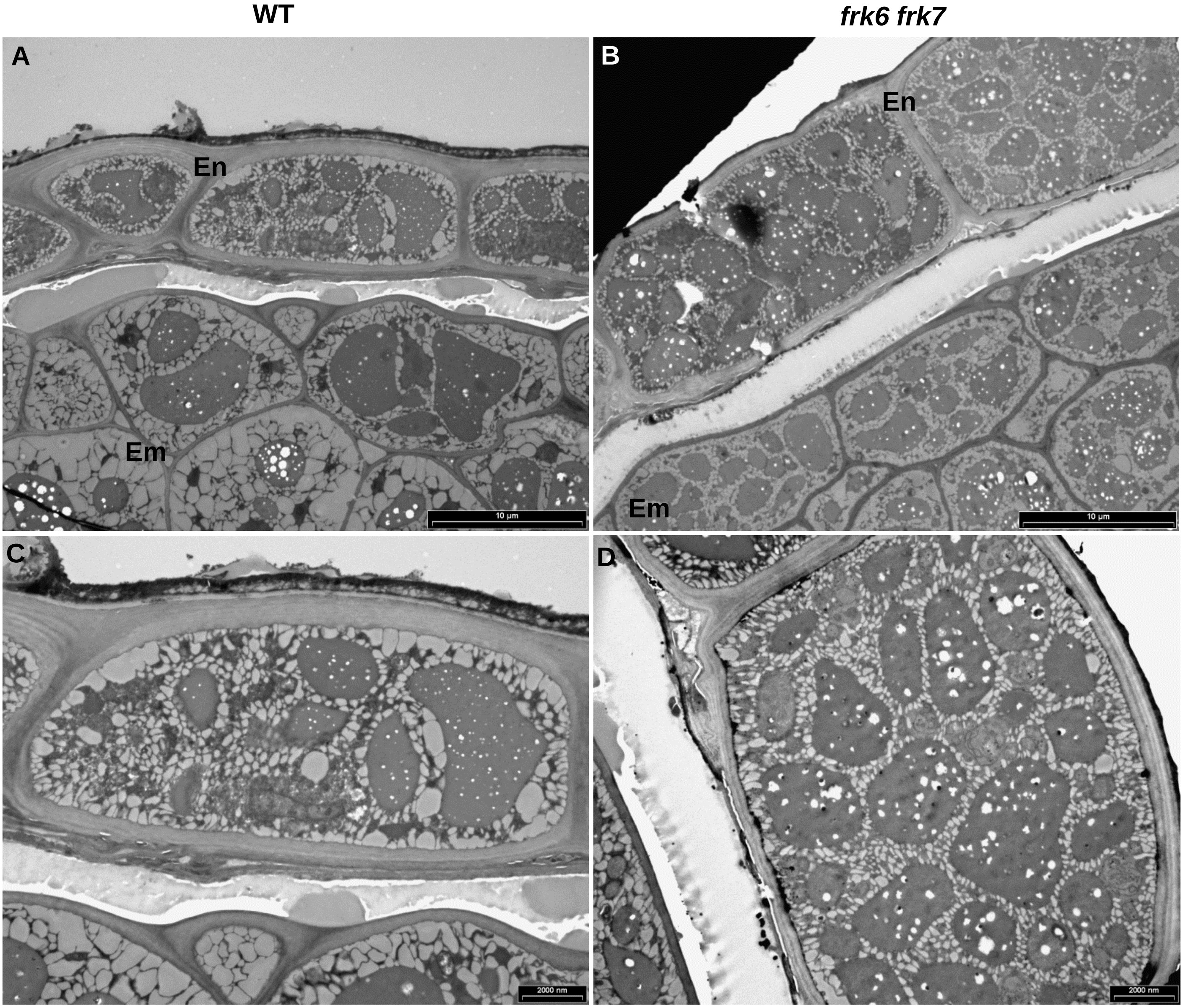

Figure S8

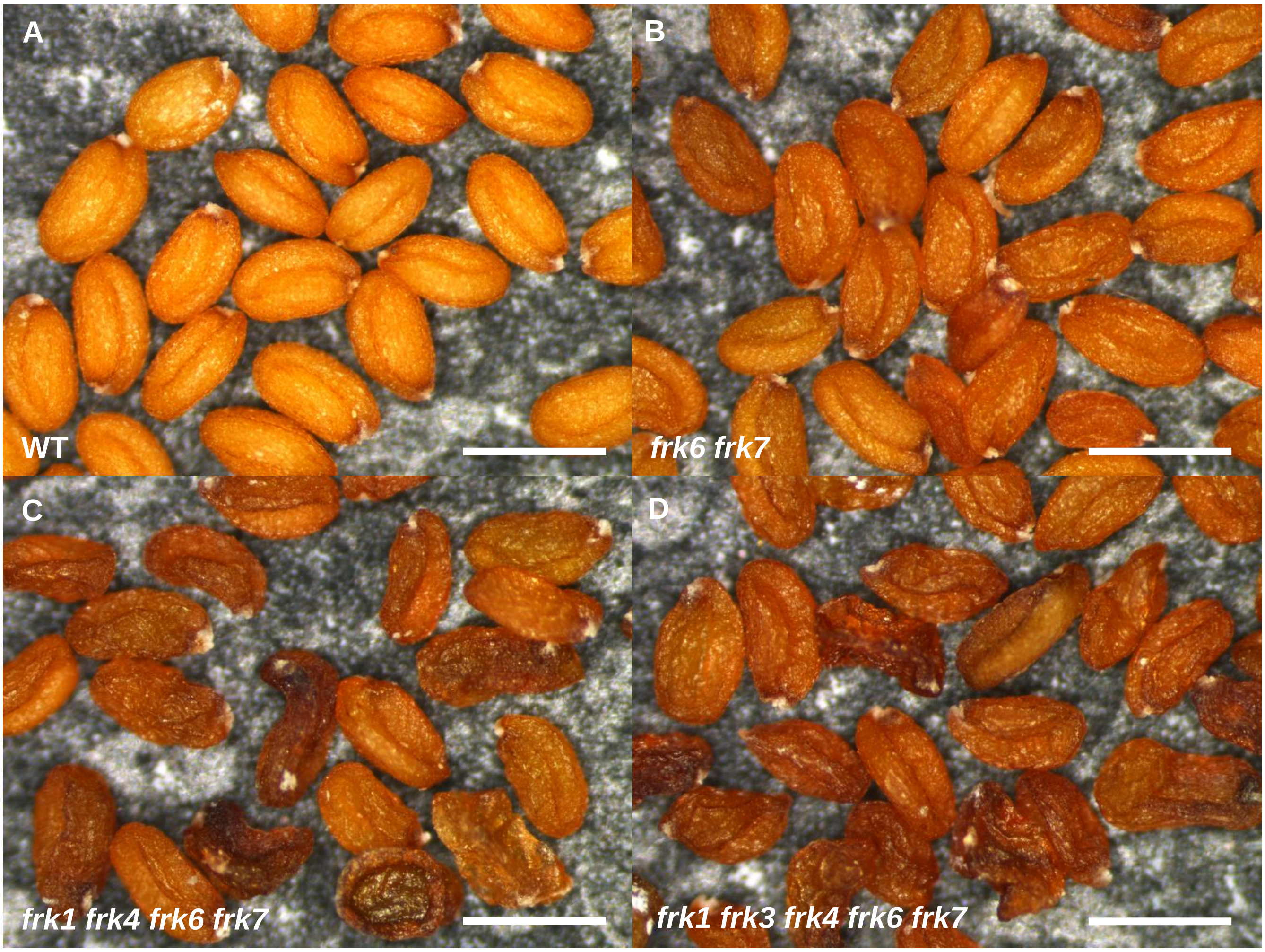
A
WT
B
frk6 frk7
C
frk1 frk4 frk6 frk7
D
frk1 frk3 frk4 frk6 frk7

Figure S9

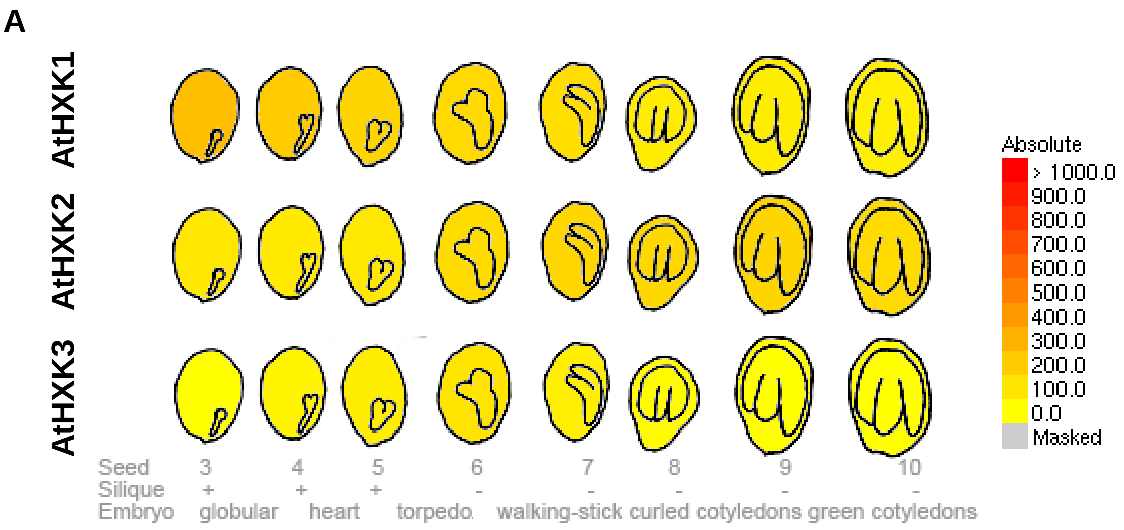

A
AtHXK1
AtHXK2
AtHXK3
Absolute
> 1000.0
900.0
800.0
700.0
600.0
500.0
400.0
300.0
200.0
100.0
0.0
Masked
Seed 3 4 5 6 7 8 9 10
Silique + + + - - - - -
Embryo globular heart torpedo walking-stick curled cotyledons green cotyledons

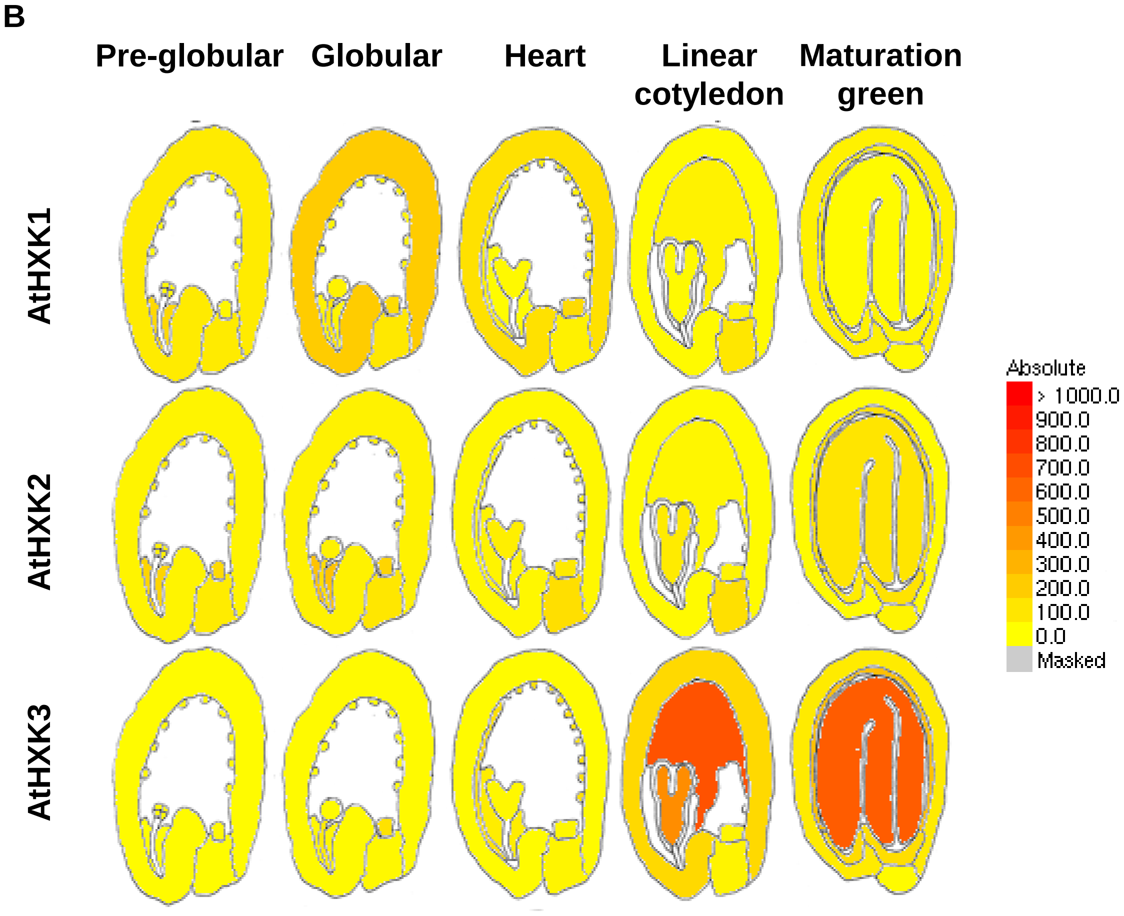

B
Pre-globular
Globular
Heart
Linear cotyledon
Maturation green
AtHXK1
AtHXK2
AtHXK3
Absolute
> 1000.0
900.0
800.0
700.0
600.0
500.0
400.0
300.0
200.0
100.0
0.0
Masked

Figure S10

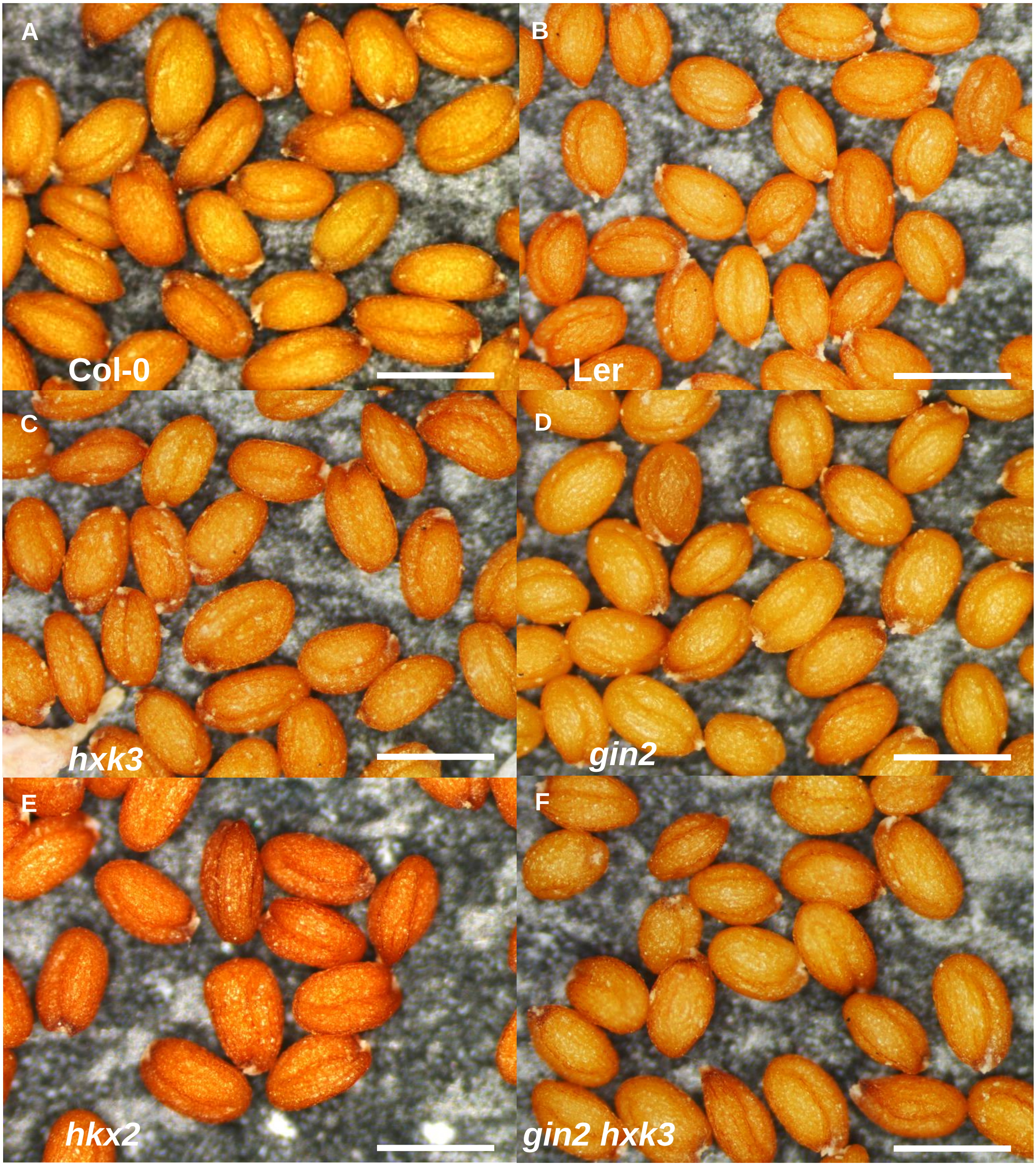

Table S1

Gene name	Gene ID	Uniprot ID	Uniprot annotation	T-DNA line	mutant name
AtFRK1	At2g31390	Q9SID0	probable FRK1	SALK_114786	*frk1*
AtFRK2	At1g06030	Q9LNE3	probable FRK2	none	
AtFRK3	At1g06020	Q9LNE4	probable FRK3	SALK_122966	*frk3*
AtFRK4	At3g59480	Q9M1B9	probable FRK4	SALK_058121	*frk4*
AtFRK5	At4g10260	O82616	probable FRK5	none	
AtFRK6	At1g66430	Q9C524	probable FRK6	SALK_142725	*frk6*
AtFRK7	At5g51830	Q9FLH8	probable FRK7	SALK_046463	*frk7*

Table S2

Primer name	Sequence (5'-3')
SALK_114786 L	TCTTACCAAAAGGCAAAACCC
SALK_114786 R	AAATTCAATAACGCGATTCCC
SALK_046463 L	CATGCGTTTGCAAATAGAAGC
SALK_046463 R	ACCAGCTTTCAAGAAAGCTCC
SALK_122966 L	CAGGAAGCTTGATGAGGAGTG
SALK_122966 R	CAAGATTCAGTTCATCTGGCC
SALK_058121 L	TCATACATGTCAAAGCTAGCC
SALK_058121 R	GCACTAGGGTTCCGGTAGAAC
SALK_142725 L	AACAACATATCAGCACTCGGG
SALK_142725 R	TTGAATGAGGTGATCGATGTG
LBb1	GCGTGGACCGCTTGCTGCAACT
At2g31390 11 F	TTCTTCTCCTTGTCCTTCCTT
At2g31390 570 R	CACTTCCATGGCCTTCAAAT
At1g66430 25 F	CGCTCTCTCAGCTTCCTCTC
At1g66430 320 R	GGAGATTCTTGTGTTGAACCATC
At5g51830 335 F	ACTTGGTGGCTCCTCTGCT
At5g51830 627 R	GGCAAGGTTCCTCAATCAAA
At1g06020 233 F	GAAAGAACGGTGTCGACGAT
At1g06020 1040 R	TTAACAAGGA GAAGCCGTAC A
At3g59480 1 F	ATTATATCCAAAACATAATCTCTCTC
At3g59480 690 R	AATCTGCTTCTGAGCCTCCT
AtHXK1 F	GCGGGAAGCAAGAGCGTGTT
AtHXK1 R	CTCCTCGGGTTGCTATGATG
SlFRK1 317 F	TAGAAATGGTGCGGTGTCAAA
SlFRK1 538 R	AACATACGGCCGAACTCATC
