# Supplementary material for: Arabidopsis Fructokinases Are Important for Seed Oil Accumulation and Vascular Development
Source: Front Plant Sci. 2017 Jan 10;7:2047. doi: 10.3389/fpls.2016.02047 (PMC5222831; doi:10.3389/fpls.2016.02047)
Supplement: Supplementary file 3 [file DataSheet1.doc]

**Legends for Supplemental Figures**

**Figure S1**  **Predicted subcellular localization of AtFRKs.**

Full-length AtFRK protein sequences were used to predict their localization. **(A)** Best subcellular localization prediction by WoLF PSORT and **(B)** subcellular localization prediction by TargetP 1.1. cTP – chloroplast transit peptide, mTP – mitochondrial transit peptide, SP – secretory pathway. Localization: C – chloroplast, _ – other.

**Figure S2 Identification of the *AtFRK* T-DNA lines.**

**(A)** Exon-intron structure and positions at which T-DNA was inserted into the *AtFRK* genes. Exons are depicted as black boxes, introns are the lines between the boxes and the 5- and 3-UTRs are shown in gray. Scale bar = 200 bp. **(B)** PCR analysis of genomic DNA to determine the homozygosity of the T-DNA insertion lines through the absence of the WT allele. Amplification was performed with right and left primers of each of the lines and AtHXK1 primers were used as a positive control. The confirmation of the T-DNA insertion in each of the lines was performed with the RP or LP for each gene and the left border primer LBb1 (not shown). Primers are listed in Table S2. **(C)** PCR analysis of cDNA to determine the presence or absence of the WT allele cDNA in the T-DNA insertion lines. AtHXK1 was used as a control. RNA from *frk1* and the double-mutant *frk6, frk7* was extracted from leaves. RNA for *frk4* was extracted the top of the inflorescence, including the shoot apical meristem and developing flowers. Primers used for the amplification of the cDNA are listed in Table S2.

**Figure S3**  **Scanning electron microscope of Arabidopsis seeds.**

WT and *frk6 frk7* double-mutant seeds were photographed using SEM. **(A)** WT seed. **(B-G)** *frk6 frk7* seeds wrinkled at various degrees. Bars – 100 μm.

**Figure S4 The *frk6 frk7* double-mutant germinated relatively slowly.**

Seeds were harvested from WT, *frk6*, *frk7* and the double-mutant plants grown under even-day conditions (12-h photoperiod) at the same time. Sixty to ninety seeds from each plant, from five individual plants per line, were surface-sterilized and sown on half-strength MS medium without any additional sugar. Following three days of stratification at 4°C, plates were transferred to a growth chamber and the germination rate was monitored by visualization of clear radicle emergence using a magnifying glass every 12 h for 4 days and after 1 week. Bars indicate standard deviation (*n* = 5). An asterisk indicates a statistical significant difference (*p* < 0.05).

**Figure S5 *AtFRKs* expression in seed development**

Analysis of the expression of AtFRKs during seed development as depicted by the BAR eFP browser based on the dataset generated by to create a gene-expression map for Arabidopsis development.

**Figure S6 *AtFRKs* expression in the different seed compartments during seed development**

Analysis of the expression of AtFRKs during seed development as depicted by the BAR eFP browser based on the dataset generated by of gene expression profiling from laser-captures of micro-dissected seeds over the course of seed development.

**Figure S7**  **Transmission electron microscope micrographs of dry seed endosperm cells.**

**(A, C)** WT. **(B, D)** *frk6 frk7* mutant. En – Endosperm, Em – Embryo. Bars: A,B - 10 µm, C,D - 2 µm.

**Figure S8**  **The seeds of the quadruple and penta mutants have more pronounced phenotype**.

Seeds were placed on black paper and photographed under a binocular microscope at maximal magnification. **(A)** Col-0 seeds, **(B)** *frk6 frk7* double-mutant seeds, **(C)** *frk1 frk4 frk6 frk7* quadruple-mutant seeds and **(D)** *frk1 frk3 frk4 frk6 frk7* penta-mutant seeds. Bars – 500 μm.

**Figure S9**  **AtHXK1-3 expression in seeds.**

Analysis of AtHXK expression during seed development as depicted by the BAR eFP browser . **(A)** Expression of AtHXKs during seed development based on the dataset generated by to create a gene-expression map for Arabidopsis development. **(B)** AtHXK expression in the different seed compartments based on the dataset created by of gene-expression profiling from laser-capture micro-dissected seeds over the course of seed development.

**Figure S10**  **The seeds of AtHXK mutants have no unusual phenotype.**

Seeds were placed on black paper and photographed under a binocular microscope at maximal magnification. **(A)** Col-0 seeds, **(B)** *Ler* seeds, **(C)** *hxk3* seeds, **(D)** *gin2* seeds, **(E)** *hxk2* and **(F)** double-mutant T3 homozygous seeds of a cross between *gin2* and *hxk3*. Bars – 500 μm.

**Table S1** *AtFRKs* gene annotation and the T-DNA mutants used in this work.

**Table S**2 List of primers used in this work.

Le, B.H., Cheng, C., Bui, A.Q., Wagmaister, J.A., Henry, K.F., Pelletier, J., et al. (2010). Global analysis of gene activity during Arabidopsis seed development and identification of seed-specific transcription factors. *Proc. Natl. Acad. Sci. U. S. A.* 107, 8063-8070.

Schmid, M., Davison, T.S., Henz, S.R., Pape, U.J., Demar, M., Vingron, M., et al. (2005). A gene expression map of *Arabidopsis thaliana* development. *Nat. Genet.* 37, 501-506.

Winter, D., Vinegar, B., Nahal, H., Ammar, R., Wilson, G.V., and Provart, N.J. (2007). An electronic fluorescent pictograph browser for exploring and analyzing large-scale biological data sets. *PLoS ONE* 2, e718.
